# The efficacy and safety of remdesivir alone and in combination with other drugs for the treatment of COVID-19: a systematic review and meta-analysis

**DOI:** 10.1186/s12879-023-08525-0

**Published:** 2023-10-09

**Authors:** Chuizhe Chen, Junde Fang, Shu Chen, Mamy Jayne Nelly Rajaofera, Xuemiao Li, Bo Wang, Qianfeng Xia

**Affiliations:** 1 Key Laboratory of Tropical Translational Medicine of Ministry of Education, NHC Key Laboratory of Tropical Disease Control, School of Tropical Medicine and The Second Affiliated Hospital, Hainan Medical University, Haikou, China; 2 Department of Pathology, Hainan General Hospital, Hainan Affiliated Hospital of Hainan Medical University, Haikou, China; 3 The First Clinical College, Hainan Medical University, Haikou, China

**Keywords:** Remdesivir, COVID-19, SARS-CoV-2, Treatment, Meta-analysis

## Abstract

**Background:**

Remdesivir is considered to be a specific drug for treating coronavirus disease 2019. This systematic review aims to evaluate the clinical efficacy and risk of remdesivir alone and in combination with other drugs.

**Research design and methods:**

The PubMed, Embase, SCIE, Cochrane Library, and American Clinical trial Center databases were searched up to 1 April 2022 to identify. Randomized controlled trials (RCTs) and observational studies comparing the efficacy of remdesivir monotherapy and combination therapy with that of control drugs.

**Results:**

Ten RCTs and 32 observational studies were included in the analysis. Regarding the primary outcome, remdesivir use reduced mortality in patients with severe COVID-19 (RR = 0.57, 95% CI (0.48,0.68)) and shortened the time to clinical improvement (MD = -2.51, 95% CI (-2.75, -2.28)). Regarding other clinical outcomes, remdesivir use was associated with improved clinical status (RR = 1.08, 95%CI (1.01, 1.17)). Regarding safety outcomes, remdesivir use did not cause liver or kidney damage (RR = 0.87, 95%CI (0.68, 1.11)) (RR = 0.88, 95%CI (0.70,1.10)). Compared with remdesivir alone, remdesivir combined with other drugs (e.g., steroids, favipiravir, and convalescent plasma) had no effect on mortality.

**Conclusion:**

The use of remdesivir can help to reduce the mortality of patients with severe COVID-19 and shorten the time to clinical improvement. There was no benefit of remdesivir combination therapy for other clinical outcomes.

**Trial registration:**

PROSPERO registration number: CRD42022322859.

**Supplementary Information:**

The online version contains supplementary material available at 10.1186/s12879-023-08525-0.

## Introduction

In December 2019, a few patients with unexplained pneumonia were found in Wuhan, Hubei Province, China [1]. After sequencing the genome of the virus from the patients’ lower respiratory tract in January 2020, it was found that the virus was novel coronavirus that, was not consistent with a known virus [2]. Subsequently, the virus was officially named SARS-CoV-2 and the pneumonia was named COVID-19 [3, 4]. As of May 27th, 2022, the number of SARS-CoV-2 infected patients worldwide has exceeded 500 million, and the death toll has reached 6.28 million [5]. Therefore, COVID-19 is a huge hazard to human health. Although some companies have launched specific drugs to treat COVID-19 [6, 7], their specific clinical efficacy still needs to be evaluated over the long term and at a large-scale to clarify the effects. Due to individual differences in drug metabolism and drug tolerance, a few COVID-19 drugs have difficulty meeting human needs to overcome SARS-CoV-2. Therefore, research on other broad-spectrum antiviral drugs is still indispensable. Remdesivir was once considered to be a specific drug for COVID-19, and it was quickly approved by the FDA, enabling its the use in the treatment of COVID-19 patients [8]. When the first COVID-19 patient in the United States was being treated, remdesivir, was already used and had a good curative effect [9]. However, several subsequent randomized controlled trial (RCT) studies showed different therapeutic effects [10, 11]. Due to various factors, the current number of RCTs evaluating the efficacy of remdesivir in treating COVID-19 is limited. Although there are some existing meta-analyses on the efficacy of remdesivir, most of these studies have predominantly included only a limited number of existing RCTs [12–16]. Consequently, the existing meta-analyses provide limited research results regarding the clinical outcomes of remdesivir, with the majority of them focusing only on a few major clinical outcomes, such as mortality, hospitalization duration, recovery rate, and adverse events [12, 13, 15–17]. Additionally, most studies have analysed remdesivir’s therapeutic effects in isolation for COVID-19 and have not evaluated its combined therapeutic effects with other drugs [12–17]. Besides RCTs, data from observational studies are also a crucial part of clinical evidence. Therefore, the analysis of observational studies is equally important. In this study, besides incorporating RCTs, we also include observational studies in the analysis to expand the sources of data. Moreover, we conduct meta-analyses not only on a few main outcomes but also assessed the impact of remdesivir treatment on additional clinical outcomes such as patient ventilator demand, clinical improvement, and organ damage (e.g., liver and kidney). Furthermore, we also evaluate the clinical outcomes of remdesivir in combination with other drugs. This comprehensive approach aims to thoroughly assess the clinical efficacy and safety of remdesivir in treating COVID-19 patients.

### Objective

The main objective of this review is to evaluate the clinical efficacy and safety of remdesivir in patients with COVID-19. In this article, we will comprehensively evaluate the efficacy and safety of remdesivir in the clinical treatment of COVID-19 patients in various aspects. This article aims to guide the current clinical use of remdesivir for the treatment of COVID-19.

## Methods

### Protocol and registration

We followed the PRISMA [18] and MOOSE [19] reporting guidelines (Additional file 1). “PROSPERO (International Prospective Register of Systematic Reviews) database” registration was performed with study number CRD42022322859.

### Inclusion and exclusion criteria

Participants must have a confirmed diagnosis of COVID-19 and be assigned to either an intervention group or a control group. The intervention groups consisted of remdesivir alone or in combination with other drugs. RCTs and observational studies were included to compare remdesivir compared with other standard care, supportive care, or placebo in the treatment of COVID-19. Review articles, case reports, case series reports, and conference reports were excluded.

### Search and selection of studies

The PubMed, Web of Science (SCIE), Embase, Cochrane Library (Trials) and American Clinical trial Center (ClinicalTrials.gov) electronic databases were searched from inception to April 01, 2022, without language restriction. The search strategy included broad search terms: “COVID-19”, “2019-nCoV”, “SARS-CoV-2”, “remdesivir” (Additional file 2).

Two investigators (Chuizhe C and Junde F) screened the data according to prespecified data collection criteria and resovled any discrepancies by consensus after discussion with two other investigators (Qianfeng X and Bo W).

### Data extraction

The studies were screened, and the following data were extracred: title, first author, time of publication, type of study, age, sex, number of cases, specific intervention measures, nationality of patients in the treatment group and the control group, and outcome.

### Quality assessment

Two reviewers (Chuizhe C and Junde F) independently assessed the quality of the selected studies according to the Cochrane Collaboration’s tool for RCTs. The results of risk of bias were graphed and assessed using Review Manager 5.4.1 [20]. We also used the Newcastle–Ottawa Scale (NOS) to assess the observational studies [21].

### Data synthesis and summary measures

Dichotomous outcome data are presented as risk ratio (RR) with 95% confidence interval (CI). Continuous outcome data were presented as mean difference (MD) with 95% CI. For the continuous outcome data without reported mean and standard deviation, but reported as interquartile range (IQR) data, we used the transformation formula given by McGrath et al. [22] to transform the continuous result data. Synthesis of data was performed using Stata 14.0. Meta-analysis pooling of RRs and MDs using the random-effects inverse-variance model. The heterogeneity among RCTs and observational studies included in the review was assessed using the I^2^ value [23]. Between-study heterogeneity can be misleadingly large when quantified by I^2^ during meta-analysis of observational studies. We evaluated the direction of effects to the GRADE guide [24] to judge the importance of heterogeneity and reflect it in GRADE. To avoid the risk of bias caused by the use of the fixed effects model under high heterogeneity, the random effects model was used when appropriate [23, 25]. Sensitivity analysis, meta-regression analysis and subgroup analysis were performed for outcomes with high heterogeneity to explore the source of heterogeneity. We performed two sensitivity analyses to assess the robustness of our research results. First, we performed a sensitivity analysis excluding studies one by one for results with high heterogeneity (I^2^ > 50%) to explore the source of the heterogeneity. Subsequently, we used Gibbs sampling, 100,000 sample iterations, and Markov chain Monte Carlo (MCMC) to conduct Bayesian meta-analysis for all the outcomes to assess the stability of the research findings. We generated funnel plots for all meta-analysis results and conducted trim and fill analysis [26] on all funnel plots. Additionally, we performed Egger’s test and Begg’s test [27, 28] on all meta-analysis results and conducted Peter’s test [29] for all binary outcomes to comprehensively assess their potential publication bias.

### Quality of evidence—GRADE Pro GDT

GRADE pro 3.6 GDT (guideline development tool) software was applied to assess the overall quality of evidence [30].

### Patient and public involvement

Patients or the public were not involved in the design, conduct, reporting, or dissemination plans of our research.

## Results

### Study flow diagram

A total of 11,505 studies were identified after electronic database searching. We removed 3792 duplicates. We screened the titles and abstracts of 7812 studies. After excluded 6980 studies, we screened the full texts of 171 articles. Finally, 42 studies, including10 RCTs and 32 observational studies, were selected for qualitative analysis [10, 11, 31–70] (Additional file 2).

### Study characteristics

The characteristics of the current systematic reviews of randomized controlled trials and observational studies are shown in Additional file 3.

### Risk of bias

**Fig. 1:**
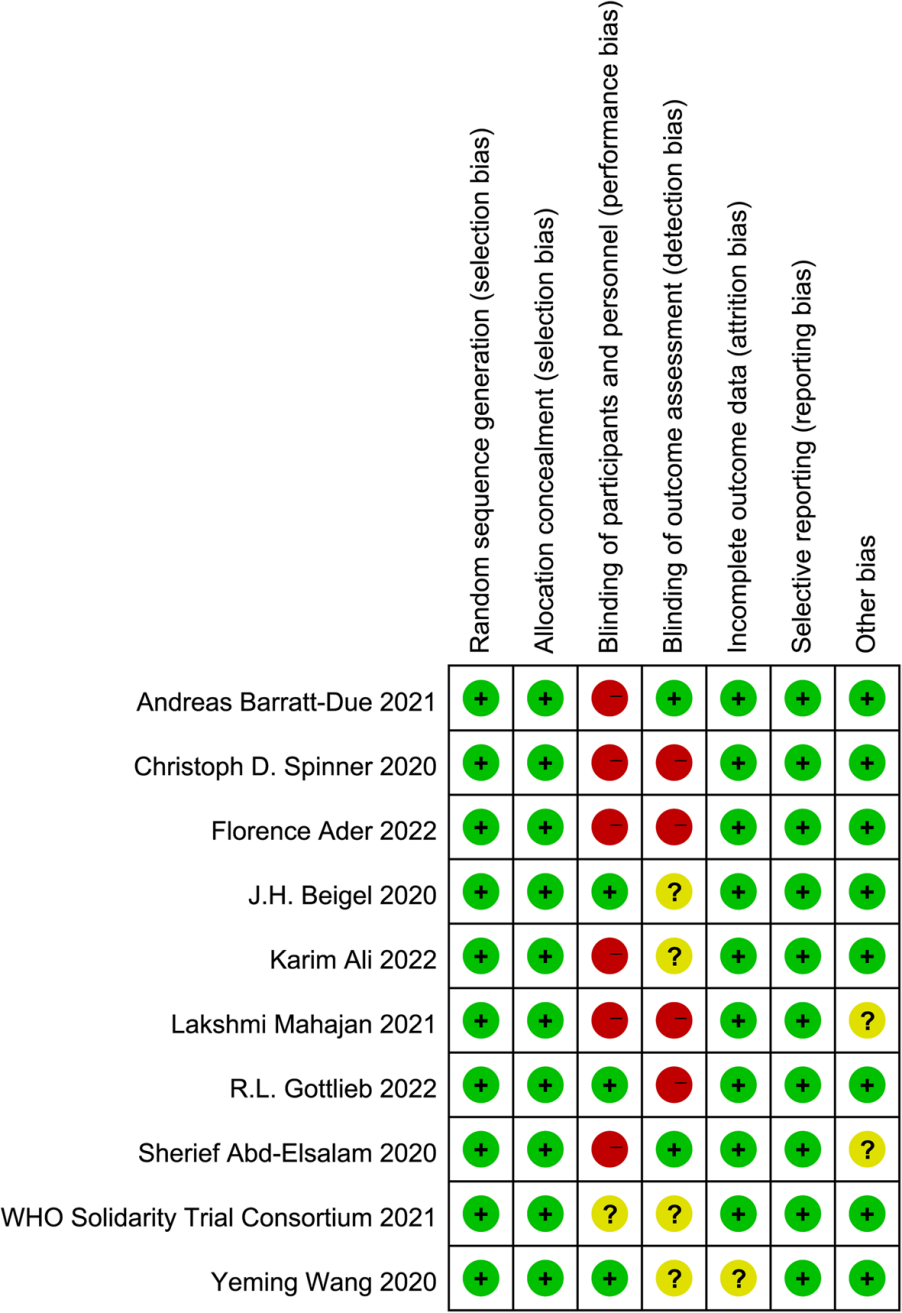
Risk of bias of RCTs summary

After screening, 42 documents were included in this analysis including 10 RCTs and 32 observational studies [10, 11, 31–70]. The details of the documents are shown in Fig. 1 and Additional file 4.

### Outcomes

#### Main outcomes of RCTs

**Fig. 2:**
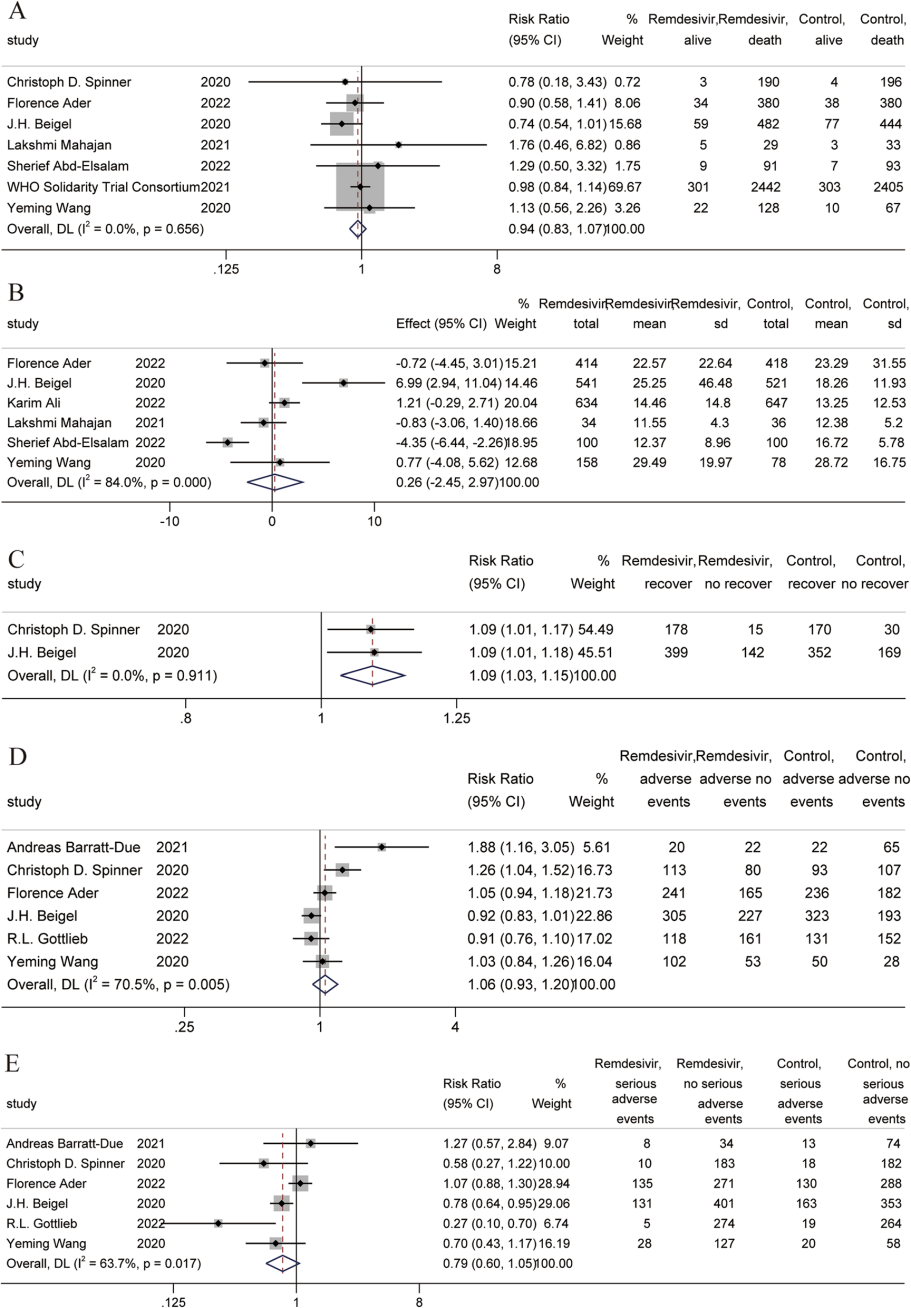
Forest plot of main outcomes in RCTs. **A** Forest plot of mortality; **B** Forest plot of duration of hospital stay; **C** Forest plot of recovery; **D** Forest plot of any adverse events; **E** Forest plot of serious adverse events

A total of 10 RCTs [10, 11, 32, 37, 39, 45, 46, 51, 52, 56] involved primary outcomes. A total of seven RCTs [10, 11, 37, 39, 46, 52, 56] involved mortality, and the results of the meta-analysis showed that the use of remdesivir did not improve mortality in COVID-19 patients [RR = 0.94, 95% CI (0.83,1.07), *P* = 0.366] (Fig. 2A). Six RCTs were included in the meta-analysis of the duration of hospital stay. The results showed that the use of remdesivir did not reduce the duration of hospital stay [MD = 0.26, 95% CI (-2.45,2.97), *P* = 0.850] (Fig. 2B). In the recovery meta-analysis, two RCTs were included. The results showed that the use of remdesivir increased the recovery rate by a small amount [RR = 1.09, 95% CI (1.03, 1.15), *P* = 0.002] (Fig. 2C). In the safety results, a total of six RCTs [10, 11, 32, 37, 39, 51] were included in the meta-analysis. The results of the meta-analysis showed that remdesivir had no effect on the incidence of any adverse events or serious adverse events (Fig. 2D and E).

#### Additional outcomes of RCTs

##### Ventilation requirements

A total of eight RCTs [10, 11, 37, 39, 45, 46, 52, 56] were included in the corresponding meta-analysis. In the meta-analysis results of ventilation requirements, the use of remdesivir was not shown to have a significant impact on changes in ventilation requirements (Additional file 5: Figure S1).

##### Other clinical results

**Fig. 3:**
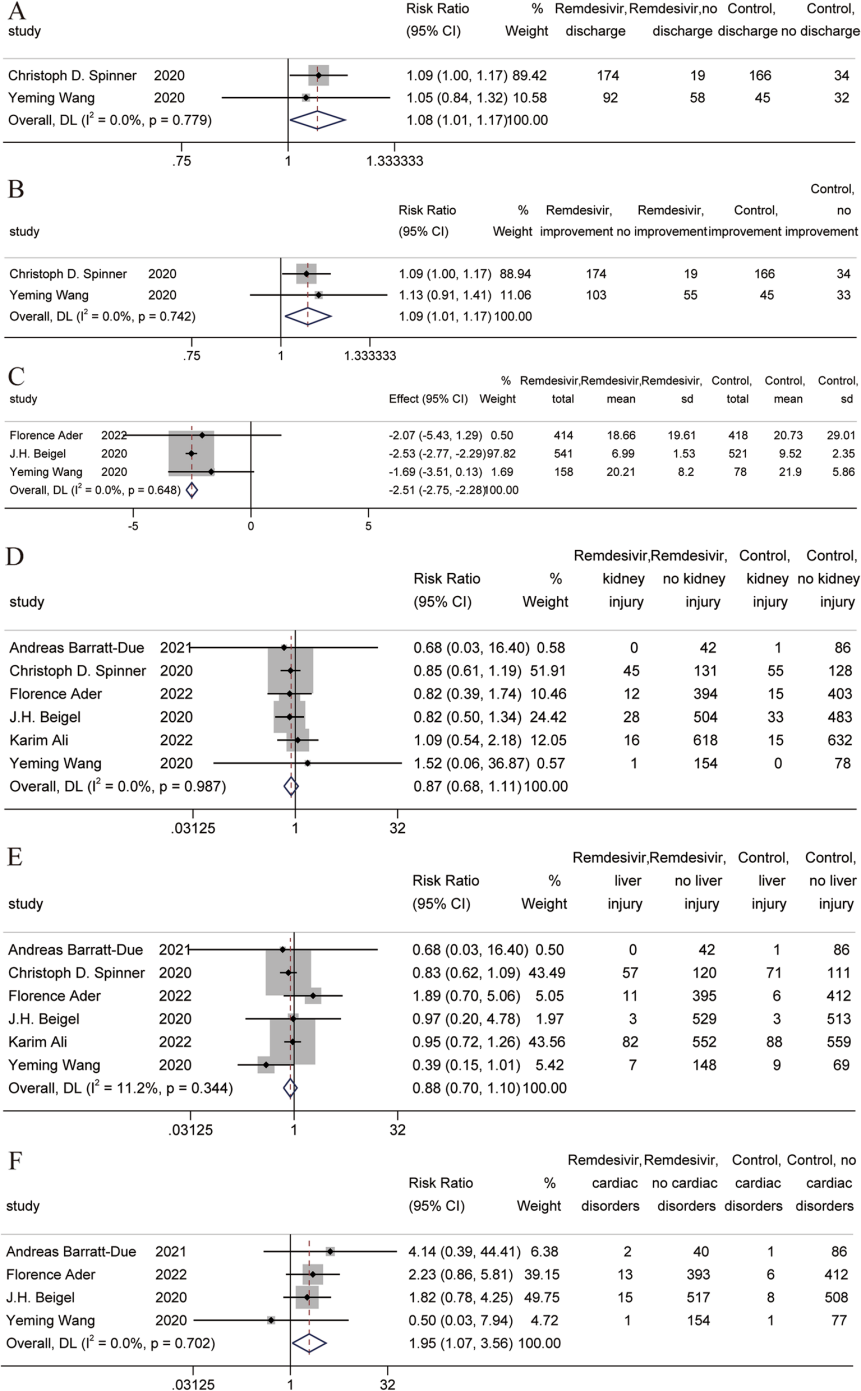
Forest plot of other clinical results in RCTs. **A** Forest plot of clinical improvement; **B** Forest plot of discharge; **C** Forest plot of time to clinical improvement; **D** Forest plot of kidney injury; **E** Forest of plot of liver injury; **F** Forest plot of cardiac disorders

A total of two RCTs [11, 37] were included in the meta-analysis of clinical improvement and discharge. The meta-analysis showed that the use of remdesivir was beneficial to clinical improvement and discharge [RR = 1.08, 95% CI (1.01, 1.17), *P* = 0.000] [RR = 1.09, 95% CI (1.01,1.17), *P* = 0.021] (Fig. 3A and B). A total of three RCTs [10, 11, 39] were included in the meta-analysis of the time to clinical improvement, which showed that the use of remdesivir helped reduce the time to clinical improvement [MD = -2.51, 95% CI (-2.75, -2.28), *P* = 0.000] (Fig. 3C). A total of six RCTs [10, 11, 32, 37, 39, 45] reported kidney and liver injury results, and meta-analysis results showed that remdesivir use did not cause kidney and liver injury [RR = 0.87, 95% CI (0.68, 1.11), *P* = 0.251] [RR = 0.88, 95% CI (0.70, 1.10), *P* = 0.272] (Fig. 3D and E). A total of four RCTs [10, 11, 32, 39] reported cardiac disorders, and meta-analysis results showed that the use of remdesivir reduced the risk of cardiac disorders [RR = 1.95, 95% CI (1.07, 3.56), *P* = 0.028] (Fig. 3F).

#### Main outcomes of the observational studies

**Fig. 4:**
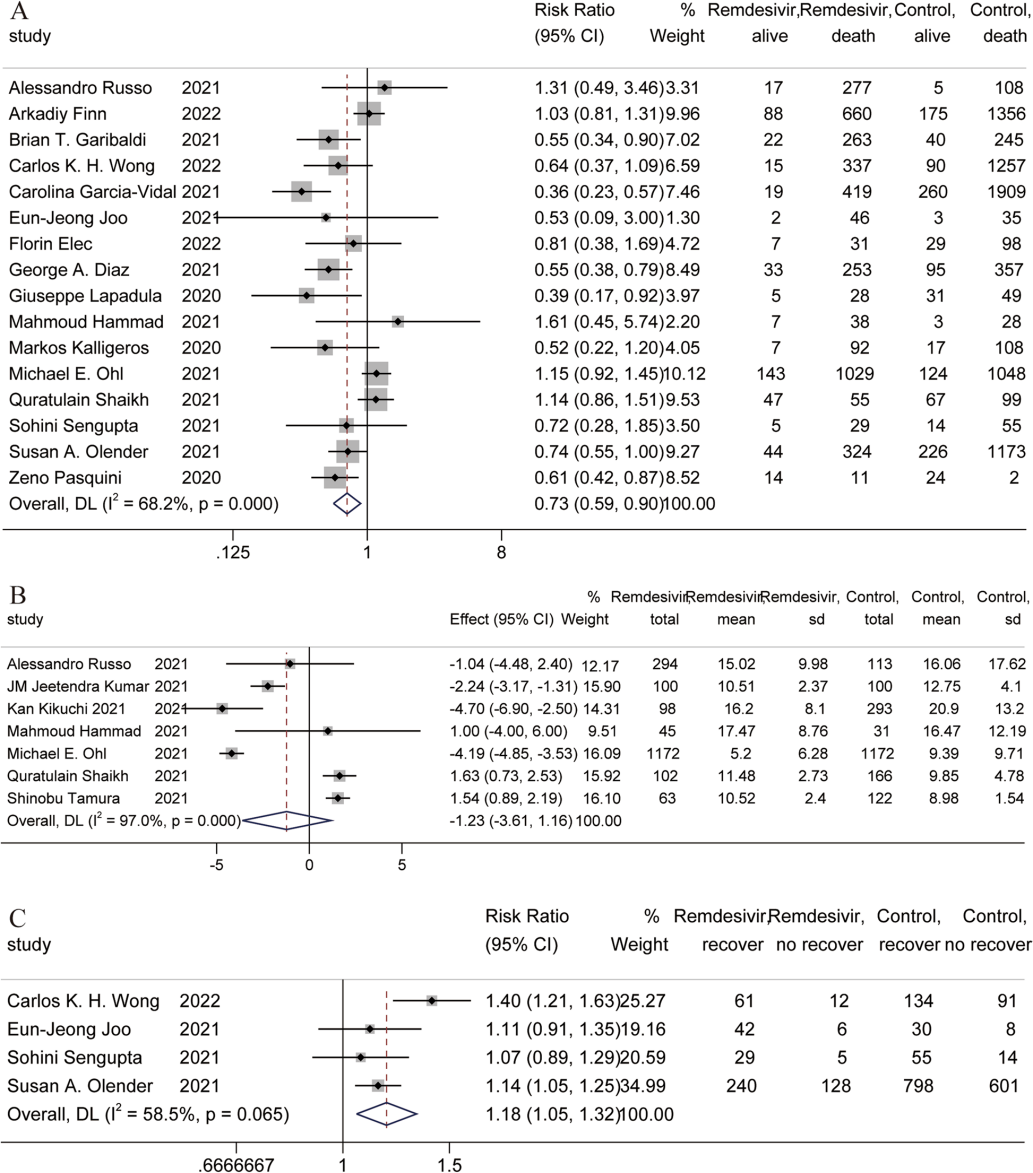
Forest plot of main outcomes in observational studies. **A** Forest plot of mortality; **B** Forest plot of duration of hospital stay; **C** Forest plot of recovery

A total of 16 studies [31, 33–36, 38, 40–42, 47–50, 54, 55, 57] reported mortality, and the meta-analysis showed that the use of remdesivir helped reduce mortality [RR = 0.73, 95% CI (0.59, 0.90), *P* = 0.003] (Fig. 4A). A total of seven studies [31, 43, 44, 47, 49, 50, 53] reported the duration of hospital stay, and the meta-analysis showed that remdesivir use had no effect on the duration of hospital stay [RR = -1.23, 95% CI (-3.61, 1.16) *P* = 0.314] (Fig. 4B). A total of four studies [35, 38, 54, 55] reported recovery, and the meta-analysis showed that the use of remdesivir helped patients recover [RR = 1.18, 95% CI (1.05, 1.32), *P* = 0.004] (Fig. 4C).

#### Additional outcomes of the observational studies

##### Ventilation requirements

A total of four studies [31, 36, 38, 40] were included in the corresponding meta-analysis. In the meta-analysis results of the duration of new use of mechanical ventilation or ECMO at baseline, the use of remdesivir did not have a significant impact on changes in the duration of new use of mechanical ventilation or ECMO at baseline (Additional file 5: Figure S2A). In a meta-analysis of new use of oxygen or low-flow oxygen at baseline, the results showed that remdesivir use increased the new use of oxygen or low-flow oxygen at baseline [RR = 1.72, 95% CI (1.48, 2.00) *P* = 0.000] (Additional file 5: Figure S2B).

##### Other clinical results

A total of 10 studies [31, 34–36, 38, 40, 42, 43, 47, 48, 50] were included in the corresponding meta-analysis. The results showed that the use of remdesivir reduced new admission to the ICU at baseline and increased clinical improvement and discharge. The use of remdesivir did not affect days to negative PCR, recovery, or kidney injury (Additional file 5: Figure S3).

#### Outcomes of remdesivir combined with other drugs

##### Outcomes of remdesivir combined with steroids

In the meta-analysis of clinical outcomes of remdesivir combined with steroids, six studies [58–63] were included in the corresponding meta-analysis. Finally, four meta-analyses were conducted. Compared with the control group, remdesivir combined with steroids showed no positive or passive significance in terms of mortality, duration of hospital stay, new admission to the ICU at baseline and liver injury in COVID-19 patients (Additional file 5: S4).

##### Outcomes of remdesivir combined with tocilizumab

In the meta-analysis of remdesivir combined with tocilizumab, three studies [54, 64, 65] were included. Compared with remdesivir or tocilizumab alone, remdesivir combined with tocilizumab significantly increased mortality [RR = 2.03, 95% CI (1.18,3.61), *P* = 0.011] (Additional file 5: Figure S5).

##### Outcomes of remdesivir with convalescent plasma

In the meta-analysis of remdesivir combined with convalescent plasma, four studies [66–69] were included. In the meta-analysis of mortality, remdesivir combined with convalescent plasma did not have a significantly different effect compared to remdesivir alone (Additional file 5: Figure S6).

##### Outcomes of remdesivir with favipiravir

In the meta-analysis of the use of remdesivir in combination with favipiravir, two studies [69, 70] were included. Remdesivir combined with favipiravir did not have a significantly different effect compared with favipiravir alone (Additional file 5: Figure S7).

#### Heterogeneity and publication bias

##### Sensitivity analysis and heterogeneity exploration

The RCT group showed heterogeneity in five outcomes, including duration of hospital stay, any adverse events, serious adverse events, new use of mechanical ventilation or ECMO at baseline, and new use of noninvasive mechanical ventilation or high-flow oxygen at baseline. After performing sensitivity analysis by excluding studies one by one, in the results of new use of noninvasive mechanical ventilation of high-flow oxygen at baseline, it was found that the heterogeneity decreased significantly after excluding the study by Spinner et al. [37] (Additional file 6: Figure S8I). Regarding the new use of noninvasive mechanical ventilation or high-flow oxygen at baseline [10, 37, 46], spinner et al. [38] had less severe disease in patients than the other two studies, and therefore disease severity may influence the need for noninvasive mechanical ventilation and high-flow oxygen distribution, leading to heterogeneity. The sensitivity analysis of other results did not find that excluding a particular study would significantly affect the heterogeneity of the meta-analysis (Additional file 6: Figure S8). In the Bayesian meta-analysis of RCT group outcomes, the results for recovery, clinical improvement, discharge, and cardiac disorders showed no effect of remdesivir administration on outcomes (Additional file 6: Table S1), refuting the results of the former meta-analysis. Therefore, we consider that these meta-analysis results are not robust. However, the results of the Bayesian meta-analysis on time to clinical improvement were consistent with the results of the former meta-analysis, and we consider that the result is robust.

There was heterogeneity in the observational study group in terms of mortality, duration of hospital stay, recovery, new use of invasive mechanical ventilation or ECMO at baseline, clinical improvement, and discharge. After sensitivity analysis, the heterogeneity of the recovery result was greatly reduced after excluding the study by Carlos K. H. Wong et al. [35] (Additional file 6: Figure S8D). The four studies [35, 38, 54, 55] were compared, and it was found that the patient population in the study by Carlos K. H. Wong et al. [35] was classified as having early mild cases, while the patient populations in the other three studies [38, 54, 55] were classified as having severe cases.

In the sensitivity analysis of the new use of mechanical ventilation of ECMO at baseline, clinical improvement and kidney injury, it was found that excluding some studies changed the heterogeneity (Additional file 6: Figure S8H, S8K and S8L). However, no significant differences were found among these studies by cross sectional comparison. In the Bayesian meta-analysis of observational study group outcomes, the results for recovery, new use of oxygen or low-flow oxygen at baseline, clinical improvement, new admission to the ICU at baseline and discharge showed no effect of remdesivir administration on outcomes (Additional file 6: Table S1), in contrast with the results of the previous meta-analysis. Therefore, we consider these meta-analysis results to be nonrobust. However, the results of the Bayesian meta-analysis of mortality of the observational study group (0.708, 95% CrI (0.553, 0.877) (Additional file 6: Table S1) were similar to the result of the former meta-analysis, and thus, we consider the results to be robust.

The meta-analysis of the duration of hospital stay and new admission to the ICU at baseline of remdesivir combined with steroids showed heterogeneity. Through sensitivity analysis, the heterogeneity was found to originate from Thomas Benfield [62] and Toshiki Kuno [63] (Additional file 6: Figure S8N and S8O). However, through horizontal comparison, no significant difference in study design and subjects of the five studies was found [58–60, 62, 63]. The meta-analysis results of the observational study group of remdesivir combined with tocilizumab showed high heterogeneity. Sensitivity analysis revealed that the source of heterogeneity was Sohini Sengupta’s study [54] (Additional file 6: Figure S8P). When comparing the three studies [64–66], no significant differences were found in study design and subjects. The result of the Bayesian meta-analysis contradicts the result of the previous meta-analysis, and thus, we consider the results to be unstable (Additional file 6: Table S1).

##### Meta-regression analysis and subgroup analysis

**Fig. 5:**
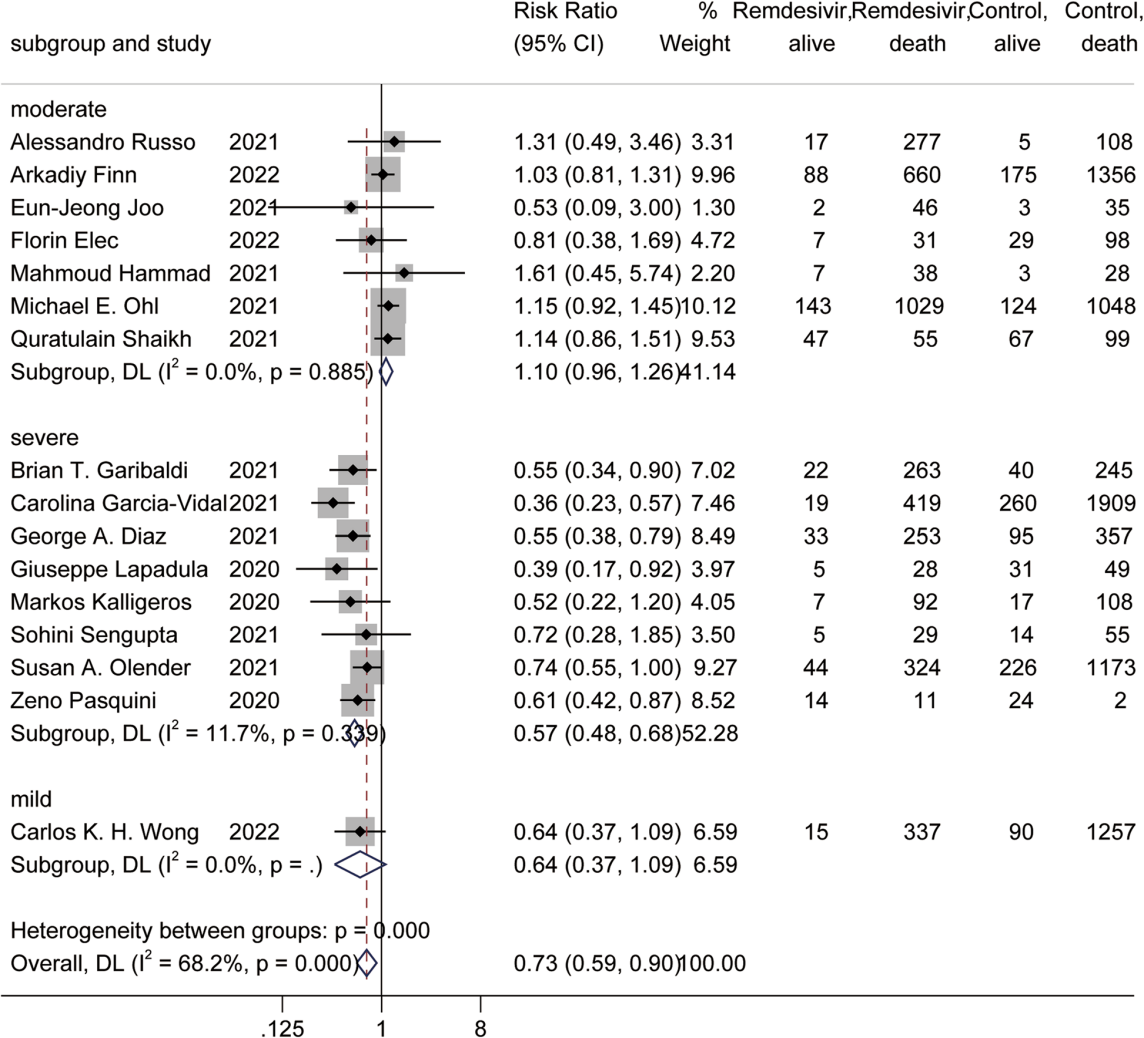
Forest plot of subgroup analysis on mortality in observational studies

Due to the small number of outcomes reported in the included RCTs. Therefore, we only performed meta-regression analysis and subgroup analysis on the mortality meta-analysis results of the observational study group [20]. In the above results, we found that remdesivir use appears to be associated with COVID-19 severity. In addition, the severity of COVID-19 is related to age [71]. Therefore, in the meta-regression, age and severity of COVID-19 patients were used as covariates. The meta-regression results showed that the severity of disease in patients with COVID-19 was associated with the use of remdesivir (regression = -0.386, *P* = 0.017) (Table 1). Then, we used the severity of the disease as the grouping standard, and divided the patients into mild group, severe group, and moderate group for subgroup analysis. In the severe group, the use of remdesivir significantly reduced mortality [RR = 0.57, 95% CI (0.48, 0.68), *P* = 0.000] by subgroup analysis (Fig. 5). In the moderate group, the use of remdesivir was not shown to have an effect. These results suggest that remdesivir use reduces mortality in severe COVID-19 patients.

**Table 1:** Meta-regression analysis

Covariates	Regression (95%CI)	*P*
Severity	-0.386 (-0.691, -0.082)	0.017
Age	-0.015 (-0.040, 0.010)	0.223

### Publication bias

We conducted Egger’s test and Begg’s test [27, 28] on all meta-analysis results, and Peter’s test [29] analysis on all binary outcomes. No significant publication bias was found (Additional file 7). However, in the results of the funnel plots, some funnel plots exhibited asymmetry (Additional file 8). After conducting trim and fill analysis on all funnel plots, we found that only the discharge of observational study [corrected RR = 1.094 95%CI (0.967,1.238)] and mortality of remdesivir combined with tocilizumab [corrected RR = 1.545 95%CI (0.947,2.521)] showed statistically significant changes in effect estimates before and after correction (Additional file 8). The remaining results did not show statistically significant changes in effect estimates before and after correction (Additional file 8). We believe that the asymmetry in the funnel plots of the discharge of observational study and mortality of remdesivir combined with tocilizumab may be attributed to potential publication bias risk and the inclusion of a limited number of studies. As for the other results, the asymmetry observed in the funnel plots may be related to the limited number of included studies [20]. Therefore, except for the discharge of observational study and mortality of remdesivir combined with tocilizumab, the remaining results have a low risk of potential publication bias and demonstrate greater stability in their findings.

### Grade analysis of outcomes

We used the GRADE to grade the results for evidence [30]. Among the “CRUCIAL” outcomes, the mortality meta-analysis results in the RCT group, and the observational study group, the quality of evidence was graded as “HIGH” and “LOW” (Additional file 9).

## Discussion

A total of 42 studies on the treatment of COVID-19 with remdesivir were included in this study. The above meta-analysis results suggest multiple benefits of remdesivir in patients with COVID-19. Among the results of these meta-analyses, some results showed high heterogeneity. In the Bayesian meta-analysis results, several results contradict the results of the former meta-analysis. After sensitivity analysis of these results, only the results of mortality and time to clinical improvement were considered robust. In addition, we performed meta-regression analysis and subgroup analysis on the results of the meta-analysis of mortality. We found that remdesivir use may be beneficial for patients with severe COVID-19. We consider that further research is likely to confirm our results. Some studies have noted that the use of remdesivir may cause liver injury, kidney injury [72], and cardiac disorders [73]. Therefore, in the safety outcomes, we also added analysis of kidney injury, liver injury and cardiac disorders. The results showed that the use of remdesivir did not affect any adverse events, serious adverse events, liver injury or kidney injury. Interestingly, in the study of Florin Elec et al. [40], kidney transplant patients were studied, and it was found that the use of remdesivir could reduce the mortality of ICU patients. The patient’s estimated glomerular filtration rate (eGFR) at discharge was improved compared with that at admission. In the study by Rita Humeniuk et al. [74], remdesivir was found to cause a temporary increase in transaminases, but this increase was reversible. Remdesivir has been found to induce cytochrome P450 enzymes (CYP1A2, CYP2B6 and CYP3A4) in human hepatocytes, which may be the reason for the transient elevation of transaminases [72]. This indicates that remdesivir may be considered for severe COVID-19 patients with liver injury and kidney injury, under strict monitoring of patients’ liver and kidney function changes.

Notably, the use of remdesivir may increase new admission to the ICU at baseline and cardiac disorders. Sensitivity analyses showed that these two findings were not robust. However, several studies have reported that the use of remdesivir may cause cardiac disorders such as abnormal QT interval and bradycardia [75, 76]. The reason for this is not yet clear, but remdesivir can be considered for use among severe COVID-19 patients with tachycardia [76]. Therefore, for patients with cardiac disorders, remdesivir needs to be used with caution.

Compared with remdesivir alone, remdesivir combined with other drugs steroids favipiravir, and convalescent plasma had no effect on mortality. The meta-analysis showed an increase in mortality with remdesivir plus tocilizumab, although sensitivity analyses and publication bias analysis showed that this result was not robust. The efficacy of tocilizumab, a recombinant humanized anti-IL-6 receptor monoclonal antibody, in the treatment of COVID-19 is not yet clear [77]. The blockade of IL-6 by tocilizumab, although some of the immune dysregulation may be rescued, may also lead to the generation of a systemic cytokine storm [78]. Therefore, caution needs to be considered when remdesivir is combined with other drugs, especially tocilizumab. The combination of remdesivir and tocilizumab is not recommended for patients with COVID-19. Given that patients receiving combined treatment with remdesivir and immunomodulators often have more severe conditions, they may require ventilation rather than antiviral therapy. Unfortunately, it is regrettable that the studies included in our analysis did not report relevant information regarding ventilation treatment. Therefore, we were unable to conduct an analysis on this aspect.

In summary, we recommend that a patient with severe COVID-19, when there is no other way to reverse the disease, is in the course of worsening. The use of remdesivir under intensive care may reduce mortality. The combination of remdesivir with other drugs is not recommended, and if it must be used, it should be done under close monitoring.

SARS-CoV-2 is prone to mutation, and there are many mutant strains, such as Beta, Delta, and Omicron [79]. There are many vaccines available, but the protection rate of vaccines is limited [80]. In addition, at present, millions of new and old infected people are waiting for treatment worldwide [5]. We still need many RCT studies to determine which drugs are highly efficient in treating COVID-19, to meet the uncertain challenges in the future. We need not only a few first-line specific drugs [6, 7] but also multiple second-line broad-spectrum antiviral drugs such as remdesivir to address the challenges of COVID-19. One strength of the current systematic review is the inclusion of observational studies to complement the insufficient number of RCTs. However, many of the included results studies had a small sample size, so the robustness of the meta-analysis results is worrisome. We performed two sensitivity analyses to assess the robustness of the results. However, some articles did not obtain the mean and standard deviation for continuous result data, and we used the conversion formula, which inevitably caused bias and reduced the level of evidence in the analysis results. Some outcomes have heterogeneity, and the source of heterogeneity was not identified. However, our grading of evidence by GRADE, as well as sensitivity analyses, meta-regression and subgroup analyses, explored some of the heterogeneity and identified sources of heterogeneity, which is another strength of this article. Furthermore, some outcomes exhibited asymmetry in the funnel plots due to the limited number of studies included. However, we conducted a comprehensive and thorough assessment to evaluate the potential risk of publication bias and the stability of results.

## Quality of evidence: (GRADE)

The overall quality of systematic review is “MODERATE”. “CRITICAL” outcomes for mortality. In the RCT group results, the quality of evidence was “HIGH”, and in the observational study, the quality of evidence was “LOW”. However, after subgroup analysis, we found that the use of remdesivir may have a mortality benefit in severe patients. This evidence suggests that further research is very likely to have an important impact on our confidence in the estimate of mortality and likely to change the estimate. Further randomized controlled trials of patients with severe COVID-19 may produce results beneficial to mortality.

## Conclusion

Evidence from our systematic review showed that remdesivir was beneficial in terms of the time to mortality of severe COVID-19 patients and the time to clinical improvement. In other respects, remdesivir had no effect on COVID-19 patients. Remdesivir is not indicated for use in combination with other drugs. Remdesivir may have some benefit in reducing mortality in severe COVID-19 patients, and the quality of evidence was “LOW”. The use of remdesivir can shorten the time to clinical improvement in COVID-19 patients, and the quality of evidence was “HIGH”. The use of remdesivir did not cause adverse reactions or increased liver and kidney damage, and the quality of evidence was “HIGH”.

### Supplementary Information

**Additional file 1.** PRISMA checklist.

**Additional file 2.** Search strategies.

**Additional file 3.** Characteristics of study.

**Additional file 4.** NOS of observational study.

**Additional file 5: Figure S1.** Forest plot of ventilation requirements (RCT). **Figure S2.** Forest plot of ventilation requirements (observational study). **Figure S3.** Forest plot of other clinical results (observational study). **Figure S4.** Forest plot of remdesivir combined with steroid. **Figure S5.** Forest plot of mortality (remdesivir with tocilizumab). **Figure S6.** Forest plot of mortality (remdesivir with convalescent plasma). **Figure S7.** Forest plot of mortality (remdesivir with favipiravir).

**Additional file 6: Figure S8.** Sensitivity analysis. **Table S1.** Bayesian Meta-analysis.

**Additional file 7.** Publication bias

**Additional file 8: Figure S9.** Funnel plot of RCTs. **Figure S10.** Funnel plot of observational studies. **Figure S11.** Funnel plot of remdesivir combined with other drugs

**Additional file 9.** GRADE evidence Profiles.

## Data Availability

The datasets used and/or analyzed during the current study are available from the corresponding author on reasonable request.
