# Supplementary material for: The efficacy and safety of remdesivir alone and in combination with other drugs for the treatment of COVID-19: a systematic review and meta-analysis
Source: BMC Infect Dis. 2023 Oct 9;23:672. doi: 10.1186/s12879-023-08525-0 (PMC10563317; doi:10.1186/s12879-023-08525-0)

Additional file 2. Search strategies

| Dadabases | Indexed and keyword terms |
| --- | --- |
| PubMed | ("covid 19"[All Fields] OR "covid 19"[MeSH Terms] OR "covid 19 vaccines"[All Fields] OR "covid 19 vaccines"[MeSH Terms] OR "covid 19 serotherapy"[All Fields] OR "covid 19 serotherapy"[Supplementary Concept] OR "covid 19 nucleic acid testing"[All Fields] OR "covid 19 nucleic acid testing"[MeSH Terms] OR "covid 19 serological testing"[All Fields] OR "covid 19 serological testing"[MeSH Terms] OR "covid 19 testing"[All Fields] OR "covid 19 testing"[MeSH Terms] OR "sars cov 2"[All Fields] OR "sars cov 2"[MeSH Terms] OR "severe acute respiratory syndrome coronavirus 2"[All Fields] OR "ncov"[All Fields] OR "2019 ncov"[All Fields] OR (("coronavirus"[MeSH Terms] OR "coronavirus"[All Fields] OR "cov"[All Fields]) AND 2019/11/01:3000/12/31[Date - Publication]) OR ("sars cov 2"[MeSH Terms] OR "sars cov 2"[All Fields] OR "2019 ncov"[All Fields]) OR ("sars cov 2"[MeSH Terms] OR "sars cov 2"[All Fields] OR "sars cov 2"[All Fields])) AND ("remdesivir"[Supplementary Concept] OR "remdesivir"[All Fields]) Filters: Publication date from 1000/01/01 to 2022/04/01 |
| Web of Science (SCIE) | (TS=(COVID-19 OR SARS-CoV-2 OR 2019-nCoV)) AND TS=(remdesivir) (1980-2022) |
| Embase | (“COVID-19”/exp OR COVID-19) OR (“SARS-CoV-2”/exp OR “SARS-CoV-2”) OR (“2019-nCoV”/exp “2019-nCoV”)) AND (“remdesivir”/exp OR “remdesivir”) (<1966-2022) |
| Cochrane Library (Trials) | (Remdesivir) AND ("SARS-CoV-2" OR "2019-nCoV" OR "COVID-19") (Word variations have been searched) in Trials (All years) |
| American Clinical trial Center (ClinicalTrials.gov) | Condition or disease: COVID-19 OR SARS-CoV-2 OR 2019-nCoV  Study type: All studies  Study Results: All studies  Sex: All  Intervention/treatment: Remdesivir |

Appendix 2. continued - Flow diagram


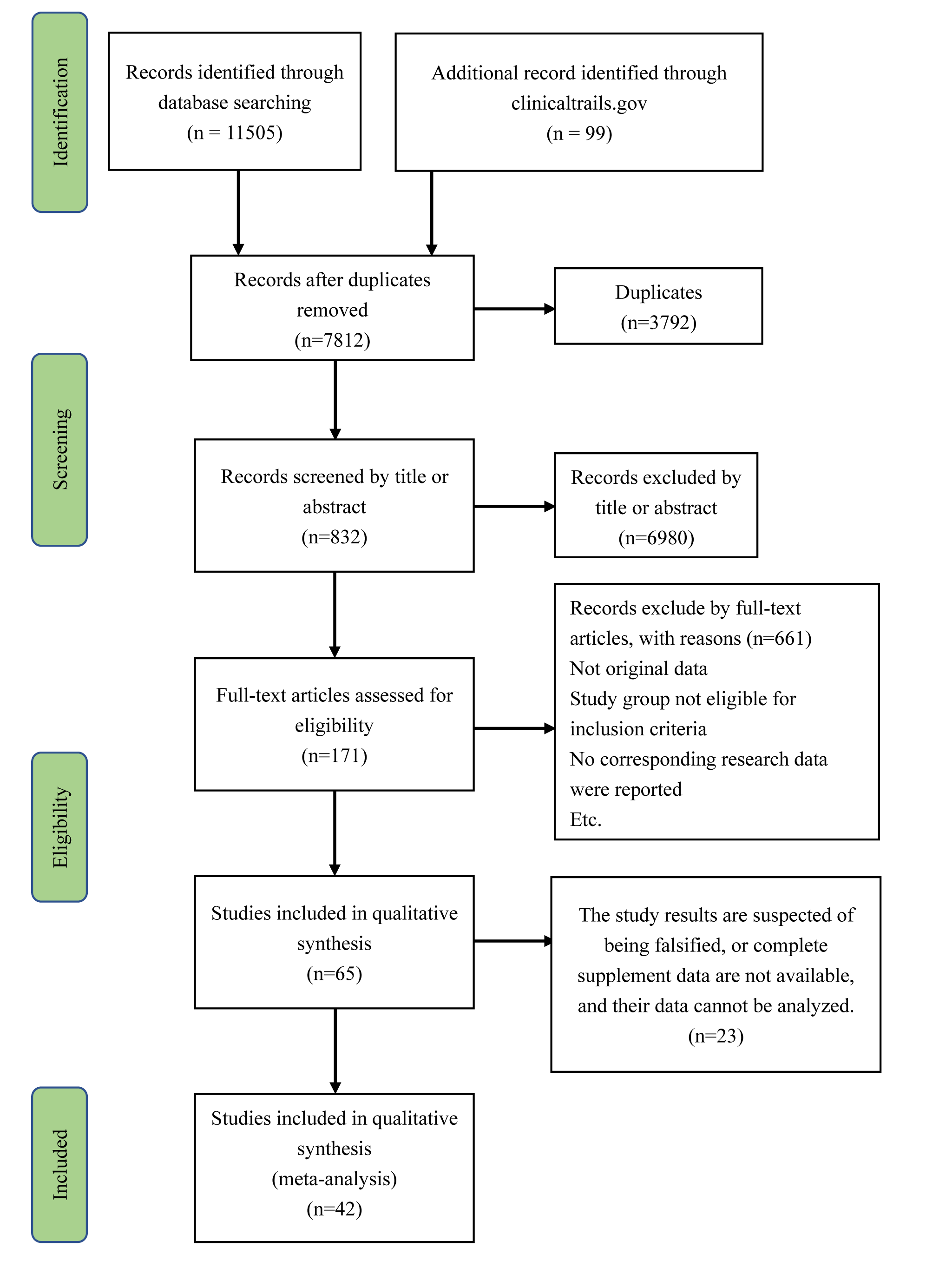

Supplement: Supplementary file 2 — Additional file 2. Search strategies. [file 12879_2023_8525_MOESM2_ESM.docx]
