# Supplementary material for: The efficacy and safety of remdesivir alone and in combination with other drugs for the treatment of COVID-19: a systematic review and meta-analysis
Source: BMC Infect Dis. 2023 Oct 9;23:672. doi: 10.1186/s12879-023-08525-0 (PMC10563317; doi:10.1186/s12879-023-08525-0)
Supplement: Supplementary file 3 — Additional file 3. Characteristics of study. [file 12879_2023_8525_MOESM3_ESM.docx]

Additional file 3. Characteristics of study

| Study | Published years | Study type ^b^ | Intervention group(n) | Control group(n) | Age  Intervention/Control (Standard Deviation or IQR) | Female Intervention/Control (%) | Race  Intervention/Control (%) | Outcomes ^d^ |
| --- | --- | --- | --- | --- | --- | --- | --- | --- |
| J.H. Beigel et al. [10] | 2020 | RCT | Remdesivir (541) | Placebo (521) | 58.6(14.6) / 59.2(15.4) | 189(34.9) / 189(36.3) | American Indian or Alaska Native: 4(0.7)/3(0.6)  Asian: 79(14.6)/56(10.7)  Black or African American: 109(20.1)/117(22.5)  White: 134(24.8)/116(22.3) |  |
| Yeming Wang et al. [11] | 2020 | RCT | Remdesivir (158) | Placebo (78) | 66(57,73) / 64(53,70) | 69(44) / 27(35) | - |  |
| Alessandro Russo et al. [22] | 2021 | Prospective cohort study | Remdesivir (294) | standard of care (113) | 63.2(15.3) / 62.5(20) | 44(20) / 43(38) | - |  |
| Andreas Barratt-Due et al. [23] | 2021 | RCT | Remdesivir (42) | standard of care (57) | 59.7(16.5) / 58.1(15.7) | 13(31) / 14(24.6) | - |  |
| Arkadiy Finn et al. [24] | 2022 | Retrospective cohort study | Remdesivir (748) | standard of care (1531) | 64.2(16.7) / 63.0(18.4) | 317(42.4) / 741(48.4) | Asian: 9(1.2)/26(1.7)  Black: 98(13.1)/259(16.9)  NHOPI: 3(0.4)/8(0.5)  Other: 219(29.3)/450(29.4)  White: 408(54.5)/776(50.7) |  |
| Brian T. Garibaldi et al. [25] | 2021 | Retrospective cohort study | Remdesivir (285) | standard of care (285) | 60(48,70) / 62(51,75) | 125(43.9) / 127(44.6) | Black: 95(33.3)/100(35.1)  Latinx: 98(34.4)/86(30.2)  White: 59(20.7)/66(23.2)  Other: 33(11.6)/33(11.6) |  |
| Carlos K. H. Wong et al. [26] | 2022 | Retrospective cohort study | Remdesivir (352) | standard of care (1347) | 65.2(13.7) / 66.7(13.7) | 154(43.8) / 609(45.2) | - |  |
| Carolina Garcia-Vidal et al. [27] | 2021 | Retrospective cohort study | Remdesivir (438) | standard of care (2169) | 65(54,77) | 162(37) / 939(43.3) | - |  |
| Christoph D. Spinner et al. ^a^ [28] | 2020 | RCT | Remdesivir (193) | standard of care (200) | 56(45,66) | 75(39) / 75(63) | White: 107(57)/112(58)  Black: 37(20)/27(14)  Asian: 31(16)/37(19)  Other: 13(7)/17(9) |  |
| Eun-Jeong Joo et al. [29] | 2021 | Retrospective cohort study | Remdesivir (48) | Supportive care (38) | 69.0(14.9) / 68.0(10.4) | 31(64.6) / 21(55.3) | - |  |
| Florence Ader et al. [30] | 2022 | RCT | Remdesivir (414) | standard of care (418) | 63(55,73) / 64(54,72) | 123(30) / 130(31) | White: 244(68)/255(70)  North African: 49(14)/61(17)  Sub-Saharan African: 30(8)/17(5)  Other: 37(10)/31(9) |  |
| Florin Elec et al. [31] | 2022 | Retrospective cohort study | Remdesivir (38) | standard of care (127) | 53(9) / 49(12) | 17(45) / 38(30) | - |  |
| George A. Diaz et al. [32] | 2021 | Retrospective cohort study | Remdesivir (286) | supportive care (452) | 61.4(16.9) / - | 124(43.4) / - | White: 150(52.4)/-  Asian, Pacific Islander: 33(11.5)/-  Black, African American: 13(4.5)/-  Hispanic, Latino: 57(19.9)/-  Other, unknown: 33(11.5)/- |  |
| Giuseppe Lapadula et al. [33] | 2020 | Prospective cohort study | Remdesivir (33) | standard of care (80) | 59(56,69) / 61(57,67) | 10(30.3) / 13(16.2) | - |  |
| JM Jeetendra Kumar et al. [34] | 2021 | Retrospective cohort Study | Remdesivir (100) | standard of care (100) | 55(14.3) / 55(14.3) | 34(34)/ 33(33) | - |  |
| Kan Kikuchi et al. [35] | 2021 | Retrospective cohort study | Remdesivir (98) | standard of care (293) | - | 31(31.6) / 83(28.3) | - |  |
| Karim Ali et al. [36] | 2022 | RCT | Remdesivir (634) | standard of care (647) | 65(53,77) / 66(54,77) | 260(41.0) / 255(39.4) | White: 269(42.4)/255(39.4)  South Asian: 90(14.2)/110(17.0)  East Asian: 40(6.3)/42(6.5)  Indigenous or First Nations: 40(6.3)/28(4.3)  Black: 20(3.2)/25(3.9)  Arab: 22(3.5)/24(3.7)  Latin American: 23(3.6)/21(3.2)  West Asian: 8(1.3)/12(1.9)  Other: 9(1.4)/14(2.2)  Not available: 119(18.8)/126(19.5) |  |
| Lakshmi Mahajan et al. [37] | 2021 | RCT | Remdesivir (34) | standard of care (36) | 58.1(12.1) / 57.4(14.1) | 13(38.3) / 9(25.0) | - |  |
| Mahmoud Hammad et al. [38] | 2021 | Prospective cohort study | Remdesivir (45) | standard of care (31) | 9(2,18) / 10(1,18) | 20(44.4) / 14(45.1) | - |  |
| Markos Kalligeros et al. [39] | 2020 | Retrospective cohort study | Remdesivir (99) | Supportive care (125) | 58(50,68) / 60(50,68) | 30(30.3) / 44(35.2) | Asian: 1(1.0)/1(0.8)  Black or African American: 10(10.1)/20(16.0)  Hispanic or Latino: 48(48.5)/50(40.0)  Other/unknown: 5(5.1)/7(5.6)  White or Caucasian: 35(35.4)/47(37.6) |  |
| Michael E. Ohl et al. [40] | 2021 | Retrospective cohort study | Remdesivir (1172) | standard of care (1172) | 66.6(14.2) / 67.5(14.1) | 71 (6.1) / 71 (6.1) | White: 693(59.1)/674(57.5)  Black: 388(33.1)/406(34.6)  Other: 27(2.3)/38(3.2)  Missing: 65(5.5)/55(4.7) |  |
| Quratulain Shaikh et al. [41] | 2021 | Prospective cohort study | Remdesivir (102) | standard of care (166) | 55.0(12.8) / 56.2(13.3) | 27(26.5) / 36(21.7) | - |  |
| R.L. Gottlieb et al. [42] | 2022 | RCT | Remdesivir (279) | Placebo (283) | 50(15) / 51(15) | 131(47.0) / 138(48.8) | White: 228(81.7)/224(79.2)  Black: 20(7.2)/22(7.8)  American Indian or Alaska Native: 15(5.4)/21(7.4)  Asian, Native Hawaiian, or Pacific Islander: 7(2.5)/7(2.5)  Hispanic or Latinx: 123(44.1)/112(39.6)  Other: 3(1.1)/2(0.7) |  |
| Sherief Abd-Elsalam et al. [43] | 2022 | RCT | Remdesivir (100) | standard of care (100) | 55.0(14.2) / 52.0(16.3) | 34(34.0) / 47(47.0) | - |  |
| Shinobu Tamura et al. [44] | 2022 | Retrospective cohort study | Remdesivir (63) | standard of care (122) | 59.6(14.7) / 44.1(20.1) | 27(42.9) / 64(52.5) | - |  |
| Sohini Sengupta et al. [45] | 2021 | Retrospective cohort study | Remdesivir (34)  Tocilizumab + Remdesivir (25) | standard of care (69)  Remdesivir (34) | <=30: 13  31-50: 32  51-70: 66  >70：17 | 48(37.5) | - |  |
| Susan A. Olender et al. [46] | 2021 | Retrospective cohort study | Remdesivir (368) | standard of care (1399) | 59.7(14.7) / 60.5(14.8) | 137(37.2) / 522(37.3) | White: 251(68.2)/949(67.9)  Asian: 42(11.4)/164(11.7)  Black or African American: 43(11.7)/171(12.2)  Other: 32(8.7)/115(8.3) |  |
| WHO Solidarity Trial Consortium ^c^ [47] | 2021 | RCT | Remdesivir (2743) | standard of care (2708) | <50: 961 / 952  50–69: 1282 / 1287  ≥70: 500 / 469 | 1037(37.8) / 983(36.3) | Europe and Canada: 715(26.1)/698(25.8)  Latin America: 470(17.1)/514(19.0)  Asia and Africa:  1558(56.8)/1496(55.2) |  |
| Zeno Pasquini et al. [48] | 2020 | Retrospective cohort study | Remdesivir (25) | standard of care (26) | 64(57,75) / 70(63,76) | 2(8.0) / 2(7.7) | - |  |
| Aldo Marrone et al. [49] | 2022 | Prospective cohort study | Remdesivir + Dexamethasone (76) | Dexamethasone (75) | 64(55,74) / 66(55,75) | 28(36.8) / 27(36) | - |  |
| Derek T. Larson et al. [50] | 2022 | Retrospective cohort study | Remdesivir + Dexamethasone (42) | supportive care (14) | 58(51,67) / 62(50,70) | 12(28.6) / 7(50) | Caucasian: 18(42.8)/6(24.0)  Black: 14(33.3)/6 (30.0)  Other/Unreported: 10(23.8)/2(14.3) |  |
| Simon B. Gressens et al. [51] | 2022 | Retrospective cohort study | Remdesivir + Dexamethasone (90) | Dexamethasone (90) | 68.6(58.8,77.9) / 67.0(58.8,77.9) | 37(41) / 35(39) | White: 58(64)/62(69)  Black: 8(9)/7(8)  Asian: 4(4)/2(2)  Other: 20(22)/19(21) |  |
| Subhadra Mandadi et al. [52] | 2022 | Retrospective cohort study | Corticosteroid + Remdesivir (74) | Corticosteroid (74) | 61.9(15.2) / 61.3(14.9) | 32(43) / 41(55) | White: 43(58)/47(64)  Black or African American: 26(35)/23(31)  Hispanic or Latino: 10(14)/10(14)  Asian: 10(14)/0(0) |  |
| Thomas Benfield et al. [53] | 2021 | Retrospective cohort study | Remdesivir + Dexamethasone (1694) | standard of care (1053) | 69(57,79) / 71(57,80) | 621(36.7) / 474(45.0) | - |  |
| Toshiki Kuno et al. [54] | 2021 | Retrospective cohort study | Corticosteroid+ Remdesivir (999) | Corticosteroid (999) | 65.9(15.8) / 65.9(15.8) | 430(43) / 431(42.9) | White: 334(33.4)/335(33.5)  African American：151(15.1)/150(15.0)  Hispanic: 215(21.5)/213(21.3)  Asian: 81(8.1)/84(8.4)  Other: 218(21.8)/217(21.7) |  |
| Nouf K. Almaghlouth et al. [55] | 2021 | Retrospective cohort study | Tocilizumab + Remdesivir (33) | Tocilizumab (80) | <30: 1(3.0)/3(3.8)  30-49: 10(30.3) / 16(20.3)  50-69: 14(42.4) / 37(46.9)  >=70: 8 (24.2) / 23(29.1) | 18(54.6) / 34(43.0) | - |  |
| Vishal Gupta et al. [56] | 2021 | Retrospective cohort study | Tocilizumab + Remdesivir (76) | Remdesivir (414) | 58.5(21) / 57(18) | 29(38.1) / 155(37.4) | - |  |
| Anna Moniuszko-Malinowska et al. [57] | 2020 | Retrospective cohort study | Convalescent Plasma + Remdesivir (25) | Remdesivir (53) | 59.5(18.8) | 32(41.0) | - |  |
| Janak Koirala et al. [58] | 2021 | Prospective cohort Study | Convalescent Plasma + Remdesivir (114) | Remdesivir (910) | 55.8(15.7) | 346(26.3) | - |  |
| Jered Arquiette et al. [59] | 2021 | Prospective cohort study | Convalescent Plasma + Remdesivir (42) | Remdesivir (11) | 50.5(40.8,65.3) / 56(54,68) | 15(35.7) / 3(27.3) | Hispanic: 28(66.7)/7(63.6)  Asian: 9(21.4)/1(9.1)  White: 1(2.4)/1(9.1)  Black: 3(7.1)/0(0)  Other: 1(2.4)/2(18.2) |  |
| M. Nasir et al. [60] | 2020 | Retrospective cohort study | Convalescent Plasma + Remdesivir (21)  Favipiravir + Remdesivir (14) | Remdesivir (22)  Favipiravir (5) | - | 13(22.5) | - |  |
| Kübra DEMİR ÖNDER et al. [61] | 2021 | Retrospective cohort study | Favipiravir + Remdesivir (17) | Favipiravir (73) | 51.6(13) / 58.4(13.7) | 6(35.3) / 25(34.2) | - |  |

Mortality, Duration of hospital stay, Recovery, Any adverse events, Serious adverse events, New use of mechanical ventilation or ECMO (extracorporeal membrane oxygenation) at baseline, Days of mechanical ventilation or ECMO during study, New use of noninvasive ventilation or high-flow oxygen at baseline, Days to negative PCR, New use of oxygen or low-flow oxygen at baseline, Days of receiving oxygen or low-flow oxygen during study, New admission to the ICU at baseline, Clinical improvement, Time to clinical improvement, Time to recovery, Discharge, Kidney injury ^e^, Liver injury ^f^, Cardiac disorders.

RCT: Randomized Controlled Trials

a: Data were analyzed at 10 days of remdesivir administration.

b: The original data were used for prospective studies, and the data after matching analysis were used for retrospective studies with matching analysis.

c: The WHO Solidarity Trial Consortium contains data from studies by Andreas Barratt-Due et al., and Karim Ali et al. Therefore, for the data duplicately published in the three studies, the data of WHO shall be taken. If no relevant data has been published by WHO, the data of the other two studies shall be taken.

d: Outcomes were taken at the end of follow-up.

e: Data were collected to report various types of kidney injury and creatinine clearance decreased.

f: Data were collected to report various types of liver injury and transaminase abnormality.
