# Supplementary material for: The efficacy and safety of remdesivir alone and in combination with other drugs for the treatment of COVID-19: a systematic review and meta-analysis
Source: BMC Infect Dis. 2023 Oct 9;23:672. doi: 10.1186/s12879-023-08525-0 (PMC10563317; doi:10.1186/s12879-023-08525-0)
Supplement: Supplementary file 4 — Additional file 4. NOS of observational study. [file 12879_2023_8525_MOESM4_ESM.docx]

Additional file 4. NOS of observational study

| Study | Selection | | | | Comparability | Outcome | | | Newcastle-Ottawa scale |
| --- | --- | --- | --- | --- | --- | --- | --- | --- | --- |
|  | 1 | 2 | 3 | 4 |  | 1 | 2 | 3 |  |
| Aldo Marrone et al. 2022 | * | * | * | * | ** | * | * | * | 9 |
| Alessandro Russo et al. 2021 | * | * | * | * | * | * | * | * | 8 |
| Anna Moniuszko-Malinowska et al. 2020 | * | * | * | * | - | * | * | * | 7 |
| Arkadiy Finn et al. 2022 | * | * | * | * | * | * | * | * | 8 |
| Brian T. Garibaldi et al. 2021 | * | * | * | * | ** | * | * | * | 9 |
| Carlos K. H. Wong et al. 2022 | * | * | * | * | ** | * | * | * | 9 |
| Carolina Garcia-Vidal et al. 2021 | * | * | * | * | * | * | * | * | 8 |
| Derek T. Larson et al. 2022 | - | * | * | * | ** | * | * | * | 8 |
| Eun-Jeong Joo et al, 2021 | * | * | * | * | ** | * | * | * | 9 |
| Florin Elec et al. 2022 | - | * | * | * | * | * | * | * | 7 |
| George A. Diaz et al. 2021 | * | * | * | * | * | * | * | * | 8 |
| Giuseppe Lapadula et al. 2020 | - | * | * | * | * | * | * | * | 7 |
| Janak Koirala et al. 2021 | - | * | * | * | * | * | * | - | 6 |
| Jered Arquiette et al. 2021 | * | * | * | * | ** | * | * | * | 9 |
| JM Jeetendra Kumar et al. 2021 | * | * | * | * | ** | * | * | * | 9 |
| Kan Kikuchi et al. 2021 | - | * | * | * | ** | * | * | * | 8 |
| Kübra DEMİR ÖNDER et al. 2021 | * | * | * | * | ** | * | * | * | 9 |
| M. Nasir et al. 2020 | * | * | * | * | - | * | * | * | 7 |
| Mahmoud Hammad et al. 2021 | - | * | * | * | * | * | * | * | 7 |
| Markos Kalligeros et al. 2020 | * | * | * | * | ** | * | * | * | 9 |
| Michael E. Ohl et al. 2021 | - | * | * | * | ** | * | * | * | 8 |
| Nouf K. Almaghlouth et al.2021 | * | * | * | * | ** | * | * | * | 9 |
| Quratulain Shaikh et al. 2021 | * | * | * | * | * | * | * | * | 8 |
| Shinobu Tamura et al. 2022 | * | * | * | * | ** | * | * | * | 9 |
| Simon B. Gressens et al. 2022 | * | * | * | * | ** | * | * | * | 9 |
| Sohini Sengupta et al. 2021 | * | * | * | * | - | * | * | * | 7 |
| Subhadra Mandadi et al. 2022 | - | * | * | * | ** | * | * | * | 8 |
| Susan A. Olender et al. 2021 | - | * | * | * | ** | * | * | * | 8 |
| Thomas Benfield et al. 2021 | * | * | * | * | * | * | * | * | 8 |
| Toshiki Kuno et al. 2021 | - | * | * | * | ** | * | * | * | 8 |
| Vishal Gupta et al. 2021 | * | * | * | * | * | * | * | * | 8 |
| Zeno Pasquini et al. 2020 | * | * | * | * | ** | * | * | * | 9 |
