# Supplementary material for: The efficacy and safety of remdesivir alone and in combination with other drugs for the treatment of COVID-19: a systematic review and meta-analysis
Source: BMC Infect Dis. 2023 Oct 9;23:672. doi: 10.1186/s12879-023-08525-0 (PMC10563317; doi:10.1186/s12879-023-08525-0)
Supplement: Supplementary file 5 — Additional file 5: Figure S1. Forest plot of ventilation requirements (RCT). Figure S2. Forest plot of ventilation requirements (observational study). Figure S3. Forest plot of other clinical results (observational study). Figure S4. Forest plot of remdesivir combined with steroid. Figure S5. Forest plot of mortality (remdesivir with tocilizumab). Figure S6. Forest plot of mortality (remdesivir with convalescent plasma). Figure S7. Forest plot of mortality (remdesivir with favipiravir). [file 12879_2023_8525_MOESM5_ESM.docx]

Additional file 5. Forest plot

Figure S1. Forest plot of ventilation requirements (RCT)


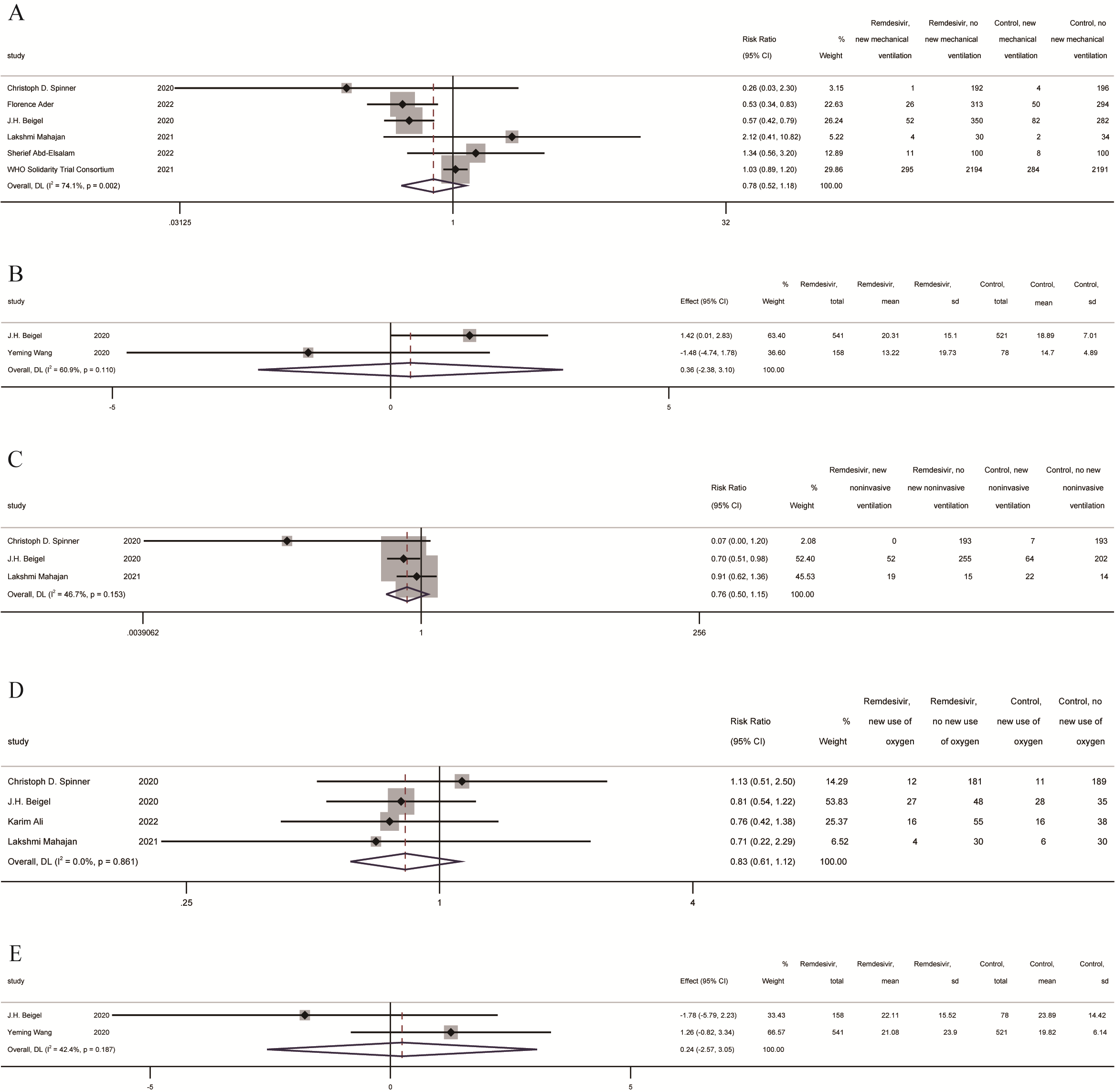


A: Forest plot of duration of new use of mechanical ventilation or ECMO at baseline; B: Forest plot of days of mechanical ventilation or ECMO during study; C: Forest plot of new use of noninvasive ventilation or high-flow oxygen at baseline; D: Forest plot of new use of oxygen or low-flow oxygen at baseline; E: Forest plot of days of receiving oxygen or low-flow oxygen during study.

Figure S2. Forest plot of ventilation requirements (observational study)


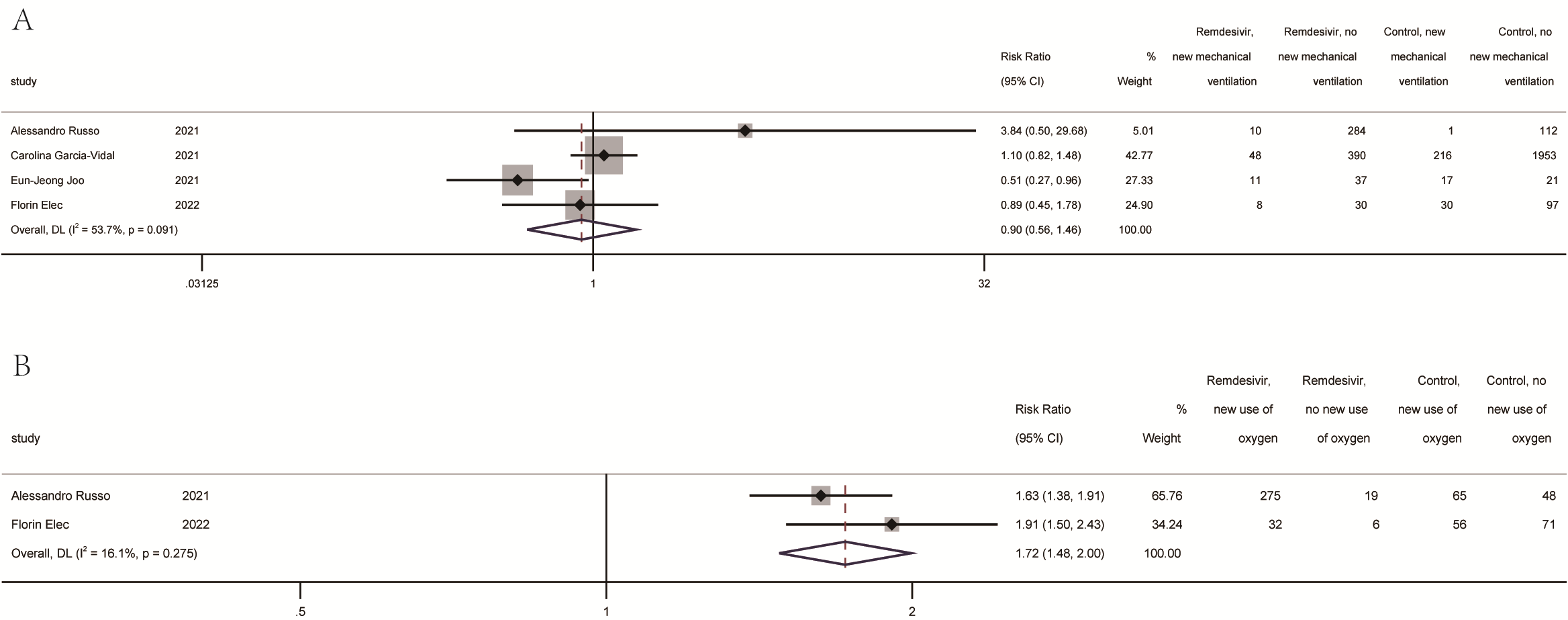


A: Forest plot of duration of new use of mechanical ventilation or ECMO at baseline; B: Forest plot of new use of oxygen or low-flow oxygen at baseline.

Figure S3. Forest plot of other clinical results (observational study)


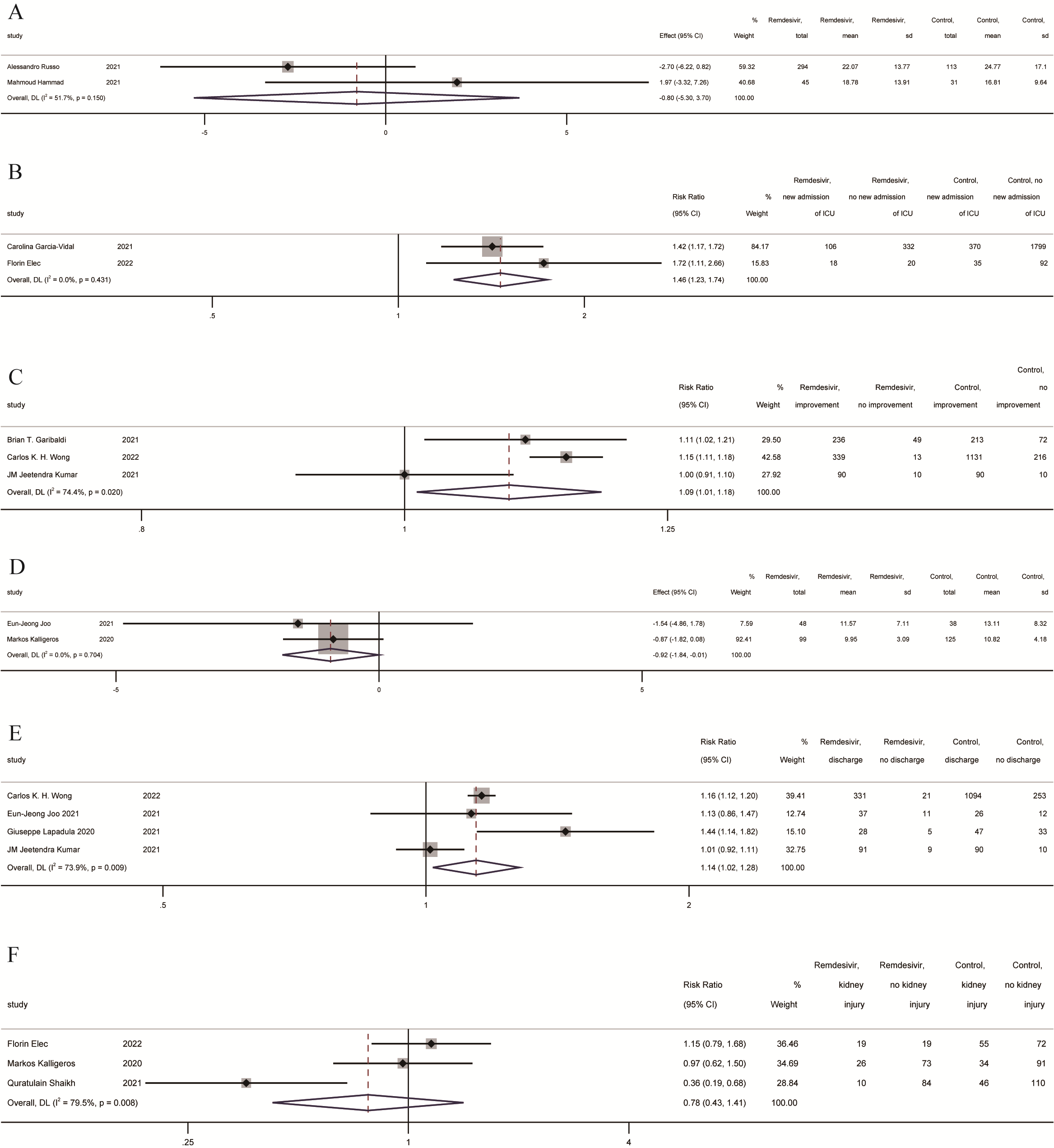


A: Forest plot of days to negative PCR; B: Forest plot of new admission to the ICU at baseline; C: Forest plot of clinical improvement; D: Forest plot of time to recovery; E: Forest plot of discharge; F: Forest plot of kidney injury.

Figure S4. Forest plot of remdesivir combined with steroid


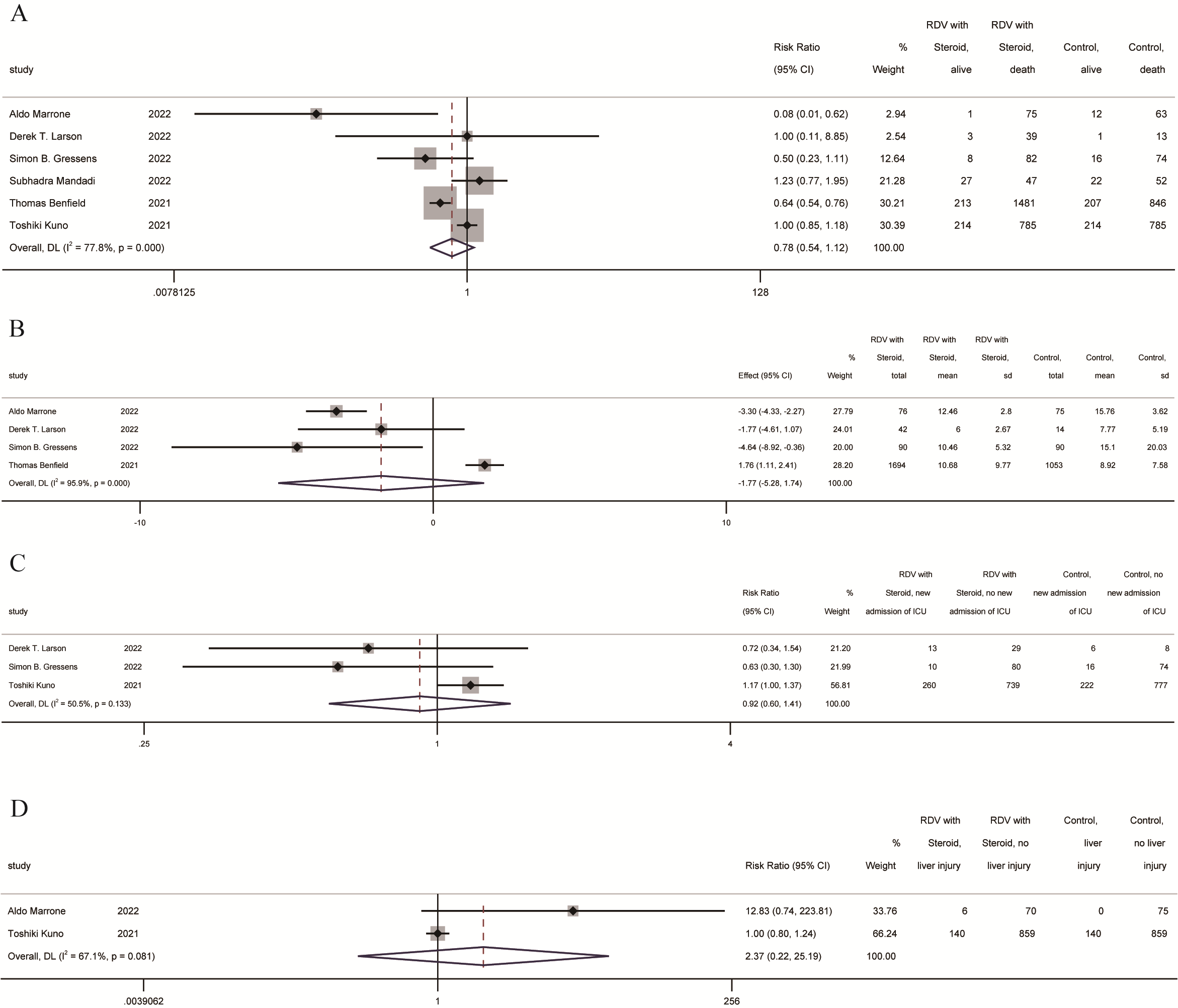


A: Forest plot of mortality; B: Forest plot of duration of hospital stay; C: Forest plot of new admission to the ICU at baseline; RDV=remdesivir

Figure S5. Forest plot of mortality (remdesivir with tocilizumab)


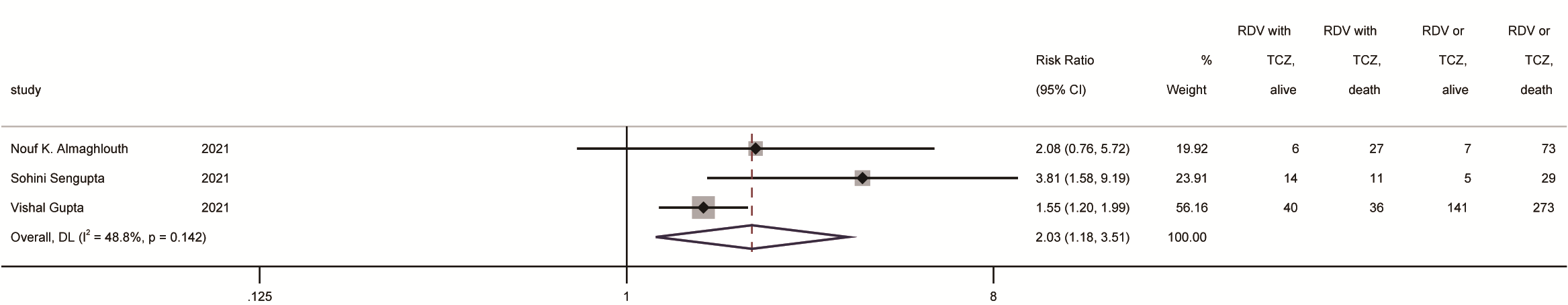


RDV=remdesivir; TCZ=tocilizumab

Figure S6. Forest plot of mortality (remdesivir with convalescent plasma)


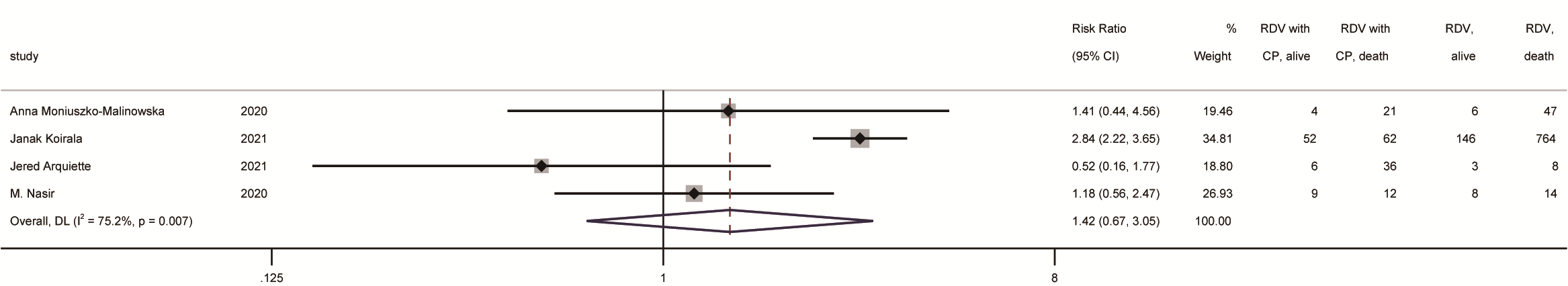


RDV=remdesivir

Figure S7. Forest plot of mortality (remdesivir with favipiravir)


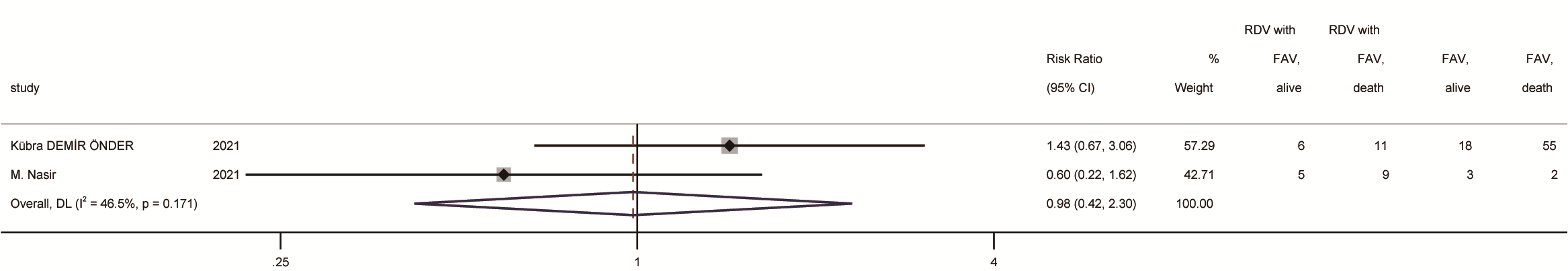


RDV=remdesivir; FAV= favipiravir
