# Supplementary material for: The efficacy and safety of remdesivir alone and in combination with other drugs for the treatment of COVID-19: a systematic review and meta-analysis
Source: BMC Infect Dis. 2023 Oct 9;23:672. doi: 10.1186/s12879-023-08525-0 (PMC10563317; doi:10.1186/s12879-023-08525-0)
Supplement: Supplementary file 6 — Additional file 6: Figure S8. Sensitivity analysis. Table S1. Bayesian Meta-analysis. [file 12879_2023_8525_MOESM6_ESM.docx]

Additional file 6. Sensitivity analysis, and Bayesian Meta-analysis

Figure S8. Sensitivity analysis


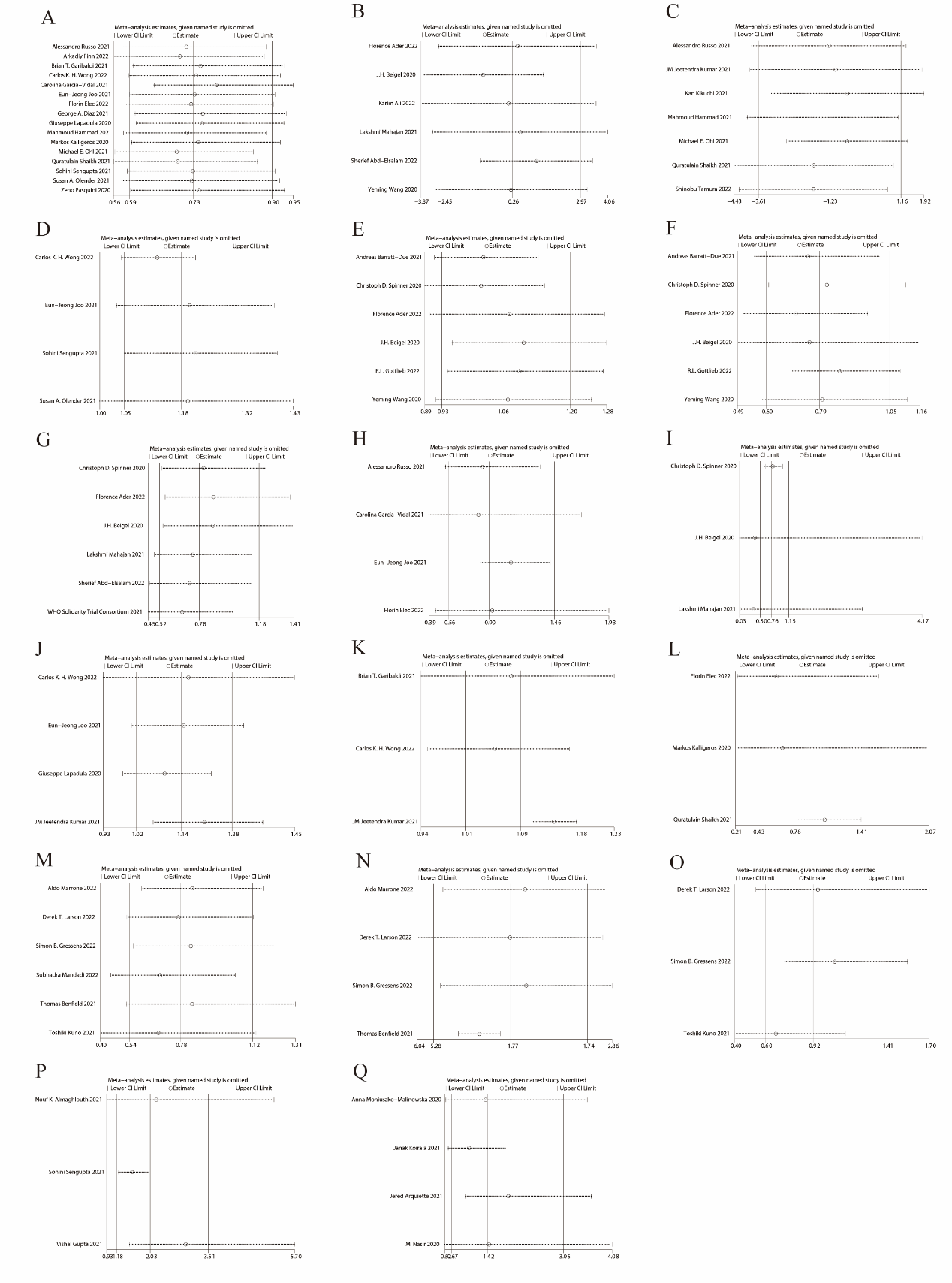


A: Sensitivity analysis of mortality (observational study); B: Sensitivity analysis of duration of hospital stay (RCT); C: Sensitivity analysis of duration of hospital stay (observational study); D: Sensitivity analysis of recovery (observational study); E: Sensitivity analysis of any adverse events (RCT); F: Sensitivity analysis of serious adverse events (RCT); G: Sensitivity analysis of new use of mechanical ventilation or ECMO at baseline (RCT); H: Sensitivity analysis of new use of mechanical ventilation of ECMO at baseline (observational study); I: Sensitivity analysis of new use of noninvasive ventilation or high-flow oxygen at baseline (RCT); J: Sensitivity analysis of clinical improvement (observational study); K: Sensitivity analysis of clinical improvement (observational study); L: Sensitivity analysis of kidney injury (observational study); M: Sensitivity analysis of mortality (remdesivir with steroid); N: Sensitivity analysis of duration of hospital stay (remdesivir with steroid); O: Sensitivity analysis of new admission to the ICU at baseline (remdesivir with steroid); P: Sensitivity analysis of mortality (remdesivir with tocilizumab); Q: Sensitivity analysis of Mortality (remdesivir with convalescent plasma).

Table S1. Bayesian Meta-analysis

| Outcome (study type) | Bayesian meta-analysis  RR/MD, (95% Credibility interval) | Rhat |
| --- | --- | --- |
| Mortality (RCT) | 0.919, 95%CrI (0.682,1.217) | 1.002 |
| Duration of hospital stay (RCT) | 0.04, 95%CrI (0.904, 1.799) | 1.002 |
| Recovery (RCT) | 1.531, 95%CrI (0.167, 5.201) | 1.001 |
| Any adverse events (RCT) | 1.069, 95%CrI (0.865, 1.363) | 1.002 |
| Serious adverse events (RCT) | 0.763, 95%CrI (0.399, 1.252) | 1.001 |
| New use of mechanical ventilation or ECMO at baseline (RCT) | 0.783, 95%CrI (0.370, 1.441) | 1.003 |
| Days of mechanical ventilation or ECMO during study (RCT) | 0.602, 95%CrI (-1.796, 2.736) | 1.001 |
| New use of noninvasive ventilation or high-flow oxygen at baseline (RCT) | 0.786, 95%CrI (0.023, 3.770) | 1.001 |
| New use of oxygen or low-flow oxygen at baseline (RCT) | 0.951, 95%CrI (0.327, 2.288) | 1.002 |
| Days of receiving oxygen or low-flow oxygen during study (RCT) | 0.287, 95%CrI (-2.274, 2.698) | 1.001 |
| Clinical improvement (RCT) | 1.693, 95%CrI (0.210, 5.445) | 1.003 |
| Time to clinical improvement (RCT) | -2.298, 95%CrI (-3.612, -0.778) | 1.001 |
| Discharge (RCT) | 1.535, 95%CrI (0.182, 6.161) | 1.001 |
| Kidney injury (RCT) | 1.133, 95%CrI (0.017, 5.735) | 1.002 |
| Liver injury (RCT) | 1.671, 95%CrI (0.018, 8.842) | 1.001 |
| Cardiac disorders (RCT) | 4.357, 95%CrI (0.121, 22.486) | 1.001 |
| Mortality (observational study) | 0.708, 95%CrI (0.553, 0.877) | 1.001 |
| Duration of hospital stay (observational study) | -1.518, 95%CrI (-3.145, 0.125) | 1.001 |
| Recovery (observational study) | 1.185, 95%CrI (0.912, 1.544) | 1.004 |
| New use of mechanical ventilation or ECMO at baseline (observational study) | 1.104, 95%CrI (0.351, 2.811) | 1.001 |
| Days to negative PCR (observational study) | -1.112, 95%CrI (-4.525, 2.447) | 1.001 |
| New use of oxygen or low-flow oxygen at baseline (observational study) | 2.851, 95%CrI (0.245, 10.191) | 1.002 |
| New admission to the ICU at baseline (observational study) | 2.441, 95%CrI (0.144, 11.325) | 1.001 |
| Clinical improvement (observational study) | 1.126, 95%CrI (0.457, 1.723) | 1.001 |
| Time to recovery (observational study) | -1.034, 95%CrI (-3.103, 0.982) | 1.001 |
| Discharge (observational study) | 1.161, 95%CrI (0.829, 1.565) | 1.005 |
| Kidney injury (observational study) | 1.143, 95%CrI (0.127, 4.176) | 1.001 |
| Mortality (remdesivir with steroid) | 0.683, 95%CrI (0.237, 1.518) | 1.001 |
| Duration of hospital stay (remdesivir with steroid) | -1.401, 95%CrI (-3.385, 0.539) | 1.003 |
| New admission to the ICU (remdesivir with steroid) | 1.059, 95%CrI (0.178, 3.418) | 1.002 |
| Liver injury (remdesivir with steroid) | 7.310, 95%CrI (0.123, 37.068) | 1.001 |
| Mortality (remdesivir with tocilizumab) | 2.589, 95%CrI (0.476, 7.631) | 1.002 |
| Mortality (remdesivir with convalescent plasma) | 2.589, 95%CrI (0.476, 7.631) | 1.001 |
| Mortality (remdesivir with favipiravir) | 2.047, 95%CrI (0.100, 8.890) | 1.001 |

Rhat is the potential scale reduction factor (at convergence, Rhat=1).
