# Supplementary material for: The efficacy and safety of remdesivir alone and in combination with other drugs for the treatment of COVID-19: a systematic review and meta-analysis
Source: BMC Infect Dis. 2023 Oct 9;23:672. doi: 10.1186/s12879-023-08525-0 (PMC10563317; doi:10.1186/s12879-023-08525-0)
Supplement: Supplementary file 7 — Additional file 7. Publication bias [file 12879_2023_8525_MOESM7_ESM.docx]

Additional file 7. Publication bias

Publication bias of Egger’s test

| Outcomes | Study | P>\|t\| (bias) |
| --- | --- | --- |
| Mortality | RCT | 0.722 |
|  | Observational study | 0.110 |
| Duration of hospital stay | RCT | 0.709 |
|  | Observational study | 0.918 |
| Recovery | RCT | - |
|  | Observational study | 0.944 |
| Any adverse events | RCT | 0.110 |
| Serious adverse events | RCT | 0.321 |
| New use of mechanical ventilation or ECMO at baseline | RCT | 0.546 |
|  | Observational study | 0.978 |
| Days of mechanical ventilation or ECMO during study | RCT | - |
| New use of noninvasive ventilation or high-flow oxygen at baseline | RCT | 0.442 |
|  | Observational study | - |
| Days to negative PCR | Observational study | - |
| New use of oxygen or low-flow oxygen at baseline | RCT | 0.822 |
|  | Observational study | - |
| Days of receiving oxygen or low-flow oxygen during study | RCT | - |
| New admission to the ICU at baseline | Observational study | - |
| Clinical improvement | RCT | - |
|  | Observational study | 0.346 |
| Time to clinical improvement | RCT | 0.810 |
| Time to recovery | Observational study | - |
| Discharge | RCT | - |
|  | Observational study | 0.993 |
| Kidney injury | RCT | 0.515 |
|  | Observational study | 0.074 |
| Liver injury | RCT | 0.938 |
| Cardiac disorders | RCT | 0.770 |
| Mortality (Remdesivir with steroid) | Observational study | 0.646 |
| Duration of hospital stay  (Remdesivir with steroid) | Observational study | 0.443 |
| New admission to the ICU at baseline (Remdesivir with steroid) | Observational study | 0.097 |
| Liver injury (Remdesivir with steroid) (Remdesivir with steroid) | Observational study | - |
| Mortality (Remdesivir with tocilizumab) | Observational study | 0.360 |
| Mortality (Remdesivir with convalescent plasma) | Observational study | 0.065 |
| Mortality (Remdesivir with favipiravir) | Observational study | - |

“-”: Data is inadequacy, cannot undertake to Egger’s test.

Publication bias of Begg’s test

| Outcomes | Study | Pr > \|z\| (continuity corrected) |
| --- | --- | --- |
| Mortality | RCT | 0.764 |
|  | Observational study | 0.893 |
| Duration of hospital stay | RCT | 0.452 |
|  | Observational study | 1.000 |
| Recovery | RCT | 1.000 |
|  | Observational study | 1.000 |
| Any adverse events | RCT | 0.133 |
| Serious adverse events | RCT | 0.707 |
| New use of mechanical ventilation or ECMO at baseline | RCT | 1.000 |
|  | Observational study | 1.000 |
| Days of mechanical ventilation or ECMO during study | RCT | 1.000 |
| New use of noninvasive ventilation or high-flow oxygen at baseline | RCT | 1.000 |
| Days to negative PCR | Observational study | 1.000 |
| New use of oxygen or low-flow oxygen at baseline | RCT | 1.000 |
|  | Observational study | 1.000 |
| Days of receiving oxygen or low-flow oxygen during study | RCT | 1.000 |
| New admission to the ICU at baseline | Observational study | 1.000 |
| Clinical improvement | RCT | 1.000 |
|  | Observational study | 0.296 |
| Time to clinical improvement | RCT | 1.000 |
| Time to recovery | Observational study | 1.000 |
| Discharge | RCT | 1.000 |
|  | Observational study | 1.000 |
| Kidney injury | RCT | 0.452 |
|  | Observational study | 0.296 |
| Liver injury | RCT | 1.000 |
| Cardiac disorders | RCT | 1.000 |
| Mortality (Remdesivir with steroid) | Observational study | 0.707 |
| Duration of hospital stay  (Remdesivir with steroid) | Observational study | 0.734 |
| New admission to the ICU at baseline (Remdesivir with steroid) | Observational study | 1.000 |
| Liver injury (Remdesivir with steroid) (Remdesivir with steroid) | Observational study | 1.000 |
| Mortality (Remdesivir with tocilizumab) | Observational study | 1.000 |
| Mortality (Remdesivir with convalescent plasma) | Observational study | 0.308 |
| Mortality (Remdesivir with favipiravir) | Observational study | 1.000 |

Publication bias of Peters’ test

| Outcomes | Study | P > \|t\| (bias) |
| --- | --- | --- |
| Mortality | RCT | 0.314 |
|  | Observational study | 0.478 |
| Recovery | RCT | - |
|  | Observational study | 0.578 |
| Any adverse events | RCT | 0.102 |
| Serious adverse events | RCT | 0.921 |
| New use of mechanical ventilation or ECMO at baseline | RCT | 0.919 |
|  | Observational study | 0.331 |
| New use of noninvasive ventilation or high-flow oxygen at baseline | RCT | - |
| New use of oxygen or low-flow oxygen at baseline | RCT | 0.173 |
|  | Observational study | - |
| New admission to the ICU at baseline | Observational study | - |
| Clinical improvement | RCT | - |
|  | Observational study | 0.343 |
| Discharge | RCT | - |
|  | Observational study | 0.457 |
| Kidney injury | RCT | 0.494 |
|  | Observational study | 0.408 |
| Liver injury | RCT | 0.161 |
| Cardiac disorders | RCT | 0.767 |
| Mortality (Remdesivir with steroid) | Observational study | 0.840 |
| New admission to the ICU at baseline (Remdesivir with steroid) | Observational study | 0.382 |
| Liver injury (Remdesivir with steroid) (Remdesivir with steroid) | Observational study | - |
| Mortality (Remdesivir with tocilizumab) | Observational study | 0.233 |
| Mortality (Remdesivir with convalescent plasma) | Observational study | 0.084 |
| Mortality (Remdesivir with favipiravir) | Observational study | - |

“-”: Data is inadequacy, cannot undertake to Peters’ test.
