# Supplementary material for: The efficacy and safety of remdesivir alone and in combination with other drugs for the treatment of COVID-19: a systematic review and meta-analysis
Source: BMC Infect Dis. 2023 Oct 9;23:672. doi: 10.1186/s12879-023-08525-0 (PMC10563317; doi:10.1186/s12879-023-08525-0)
Supplement: Supplementary file 8 — Additional file 8: Figure S9. Funnel plot of RCTs. Figure S10. Funnel plot of observational studies. Figure S11. Funnel plot of remdesivir combined with other drugs [file 12879_2023_8525_MOESM8_ESM.docx]

Additional file 8. Funnel plot

Figure S9. Funnel plot of RCTs


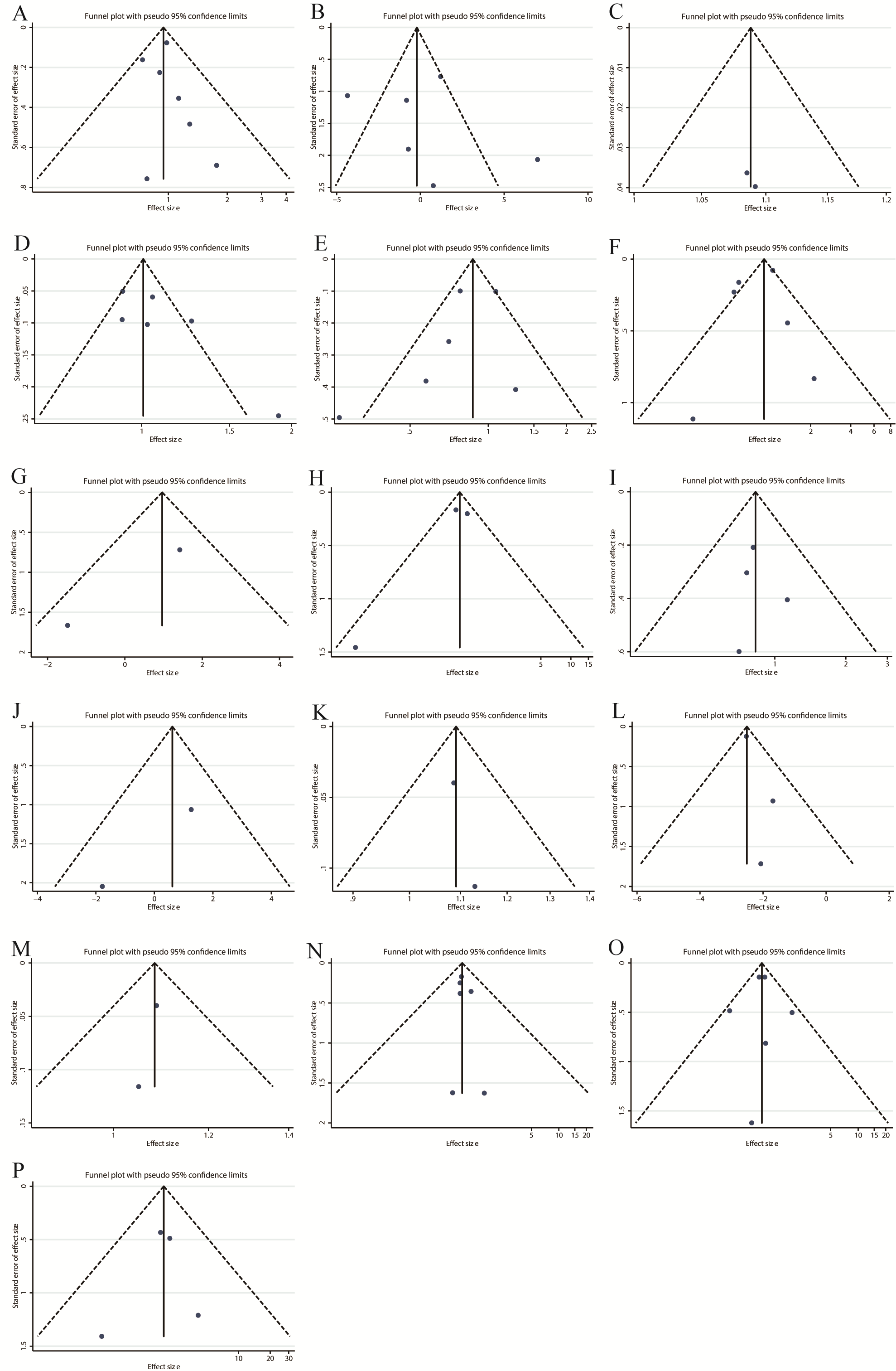


A: Funnel plot of mortality; B: Funnel plot of duration of hospital stay; C: Funnel plot of recovery; D: Funnel plot of any adverse events; E: Funnel plot of serious adverse events; F: Funnel plot of new use of mechanical ventilation or ECMO (extracorporeal membrane oxygenation) at baseline; G: Funnel plot of days of mechanical ventilation or ECMO during study; H: Funnel plot of new use of noninvasive ventilation or high-flow oxygen at baseline; I: Funnel plot of new use of oxygen or low-flow oxygen at baseline; J: Funnel plot of days of receiving oxygen or low-flow oxygen during study; K: Funnel plot of clinical improvement; L: Funnel plot of time to clinical improvement; M: Funnel plot of discharge; N: Funnel plot of kidney injury; O: Funnel plot of liver injury; P: Funnel plot of cardiac disorders.

Figure S10. Funnel plot of observational studies


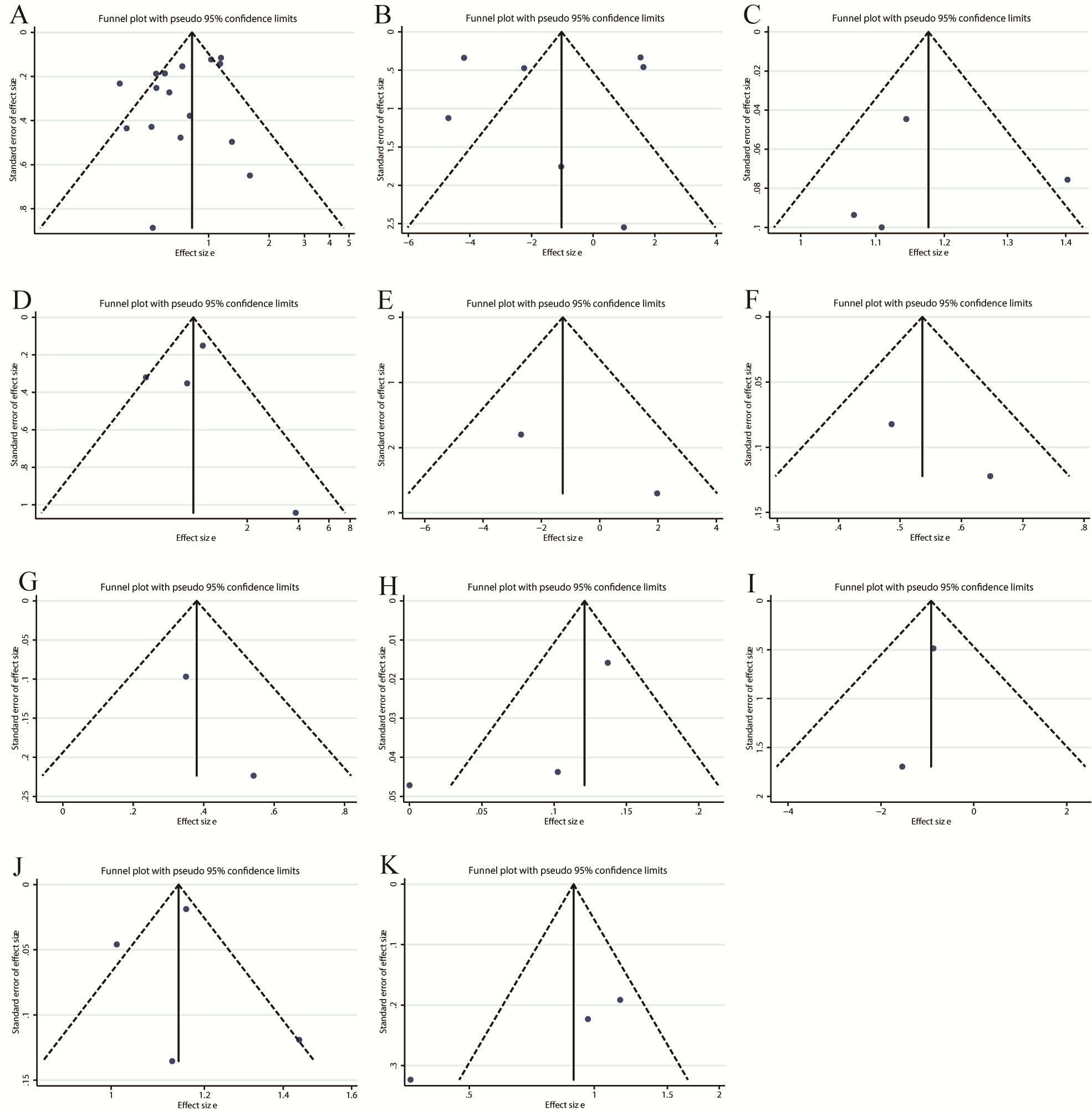


A: Funnel plot of mortality; B: Funnel plot of duration of hospital stay; C: Funnel plot of recovery; D: Funnel plot of serious adverse events; F: Funnel plot of new use of mechanical ventilation or ECMO (extracorporeal membrane oxygenation) at baseline; E: Funnel plot of days to negative PCR; F: Funnel plot of new use of noninvasive ventilation or high-flow oxygen at baseline; G: Funnel plot of new admission to the ICU at baseline; H: Funnel plot of clinical improvement; I: Funnel plot of time to clinical improvement; J: Funnel plot of discharge; K: Funnel plot of kidney injury.

Figure S11. Funnel plot of remdesivir combined with other drugs


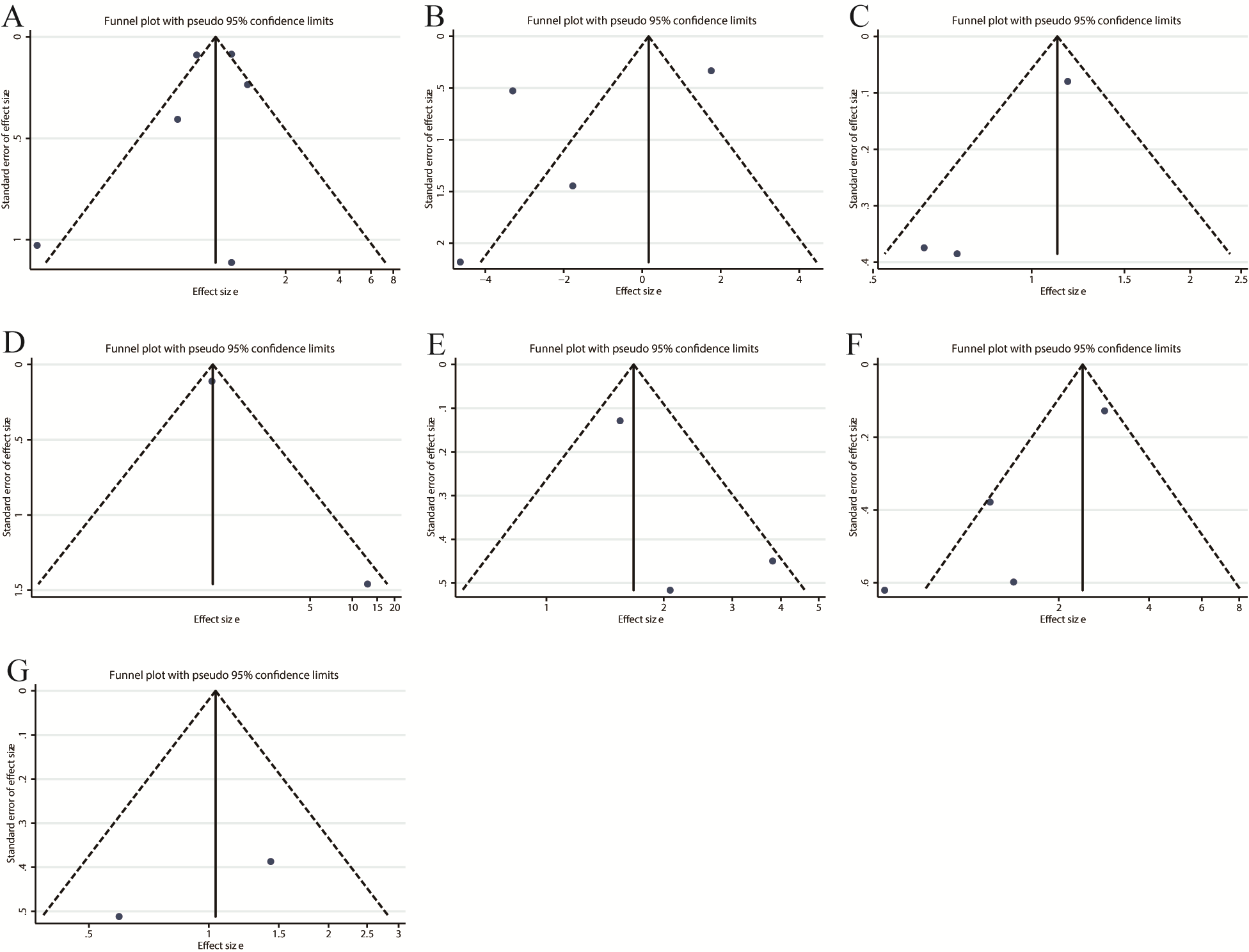


A: Funnel plot of mortality (remdesivir with steroid); B: Funnel plot of duration of hospital stay (remdesivir with steroid); C: Funnel plot of new admission to the ICU at baseline (remdesivir with steroid); D: Funnel plot of liver injury (remdesivir with steroid); E: Funnel plot of mortality (remdesivir with tocilizumab); F: Funnel plot of mortality (remdesivir with convalescent plasma); G: Funnel plot of mortality (remdesivir with favipiravir)

(Continued) Trim and fill analysis

| Outcomes | Study | uncorrected RR/MD (95%CI) | corrected RR/MD (95%CI) |
| --- | --- | --- | --- |
| Mortality | RCT | 0.944 (0.832,1.070) | 0.939 (0.828,1.064) |
|  | Observational study | 0.731 (0.593,0.902) | 0.731 (0.593,0.902) |
| Duration of hospital stay | RCT | 0.262 (-2.446,2.971) | 0.262 (-2.446, 2.971) |
|  | Observational study | -1.227 (-3.615,1.161) | -1.227 (-3.615,1.161) |
| Recovery | RCT | 1.088 (1.032,1.147) | 1.088 (1.032,1.147) |
|  | Observational study | 1.181 (1.053,1.323) | 1.181 (1.053,1.323) |
| Any adverse events | RCT | 1.055 (0.928,1.200) | 1.055 (0.928,1.200) |
| Serious adverse events | RCT | 0.793 (0.598,1.051) | 0.793 (0.598,1.051) |
| New use of mechanical ventilation or ECMO at baseline | RCT | 0.782 (0.519,1.176) | 0.782 (0.519,1.176) |
|  | Observational study | 0.902 (0.557,1.461) | 0.829 (0.493,1.391) |
| Days of mechanical ventilation or ECMO during study | RCT | 0.359 (-2.379,3.097) | 0.359 (-2.379,3.097) |
| New use of noninvasive ventilation or high-flow oxygen at baseline | RCT | 0.756 (0.498,1.147) | 0.756 (0.498,1.147) |
| Days to negative PCR | Observational study | -0.800 (-5.297,3.696) | -2.700 (-7.354,1.954) |
| New use of oxygen or low-flow oxygen at baseline | RCT | 0.829 (0.614,1.119) | 0.829 (0.614,1.119) |
|  | Observational study | 1.718 (1.480,1.995) | 1.718 (1.480,1.995) |
| Days of receiving oxygen or low-flow oxygen during study | RCT | 0.244 (-2.567,3.054) | 0.244 (-2.567,3.054) |
| New admission to the ICU at baseline | Observational study | 1.462 (1.229,1.741) | 1.462 (1.229,1.741) |
| Clinical improvement | RCT | 1.091 (1.013,1.174) | 1.091 (1.013,1.174) |
|  | Observational study | 1.093 (1.010,1.182) | 1.093 (1.010,1.182) |
| Time to clinical improvement | RCT | -2.514 (-2.750,-2.277) | -2.530 (-2.764,-2.296) |
| Time to recovery | Observational study | -0.921 (-1.837,-0.005) | -0.921 (-1.837,-0.005) |
| Discharge | RCT | 1.082 (1.005,1.165) | 1.082 (1.005,1.165) |
|  | Observational study | 1.141 (1.018,1.279) | 1.094 (0.967,1.238) |
| Kidney injury | RCT | 0.868 (0.682,1.105) | 0.868 (0.682,1.105) |
|  | Observational study | 0.776 (0.425,1.415) | 0.776 (0.425,1.415) |
| Liver injury | RCT | 0.881 (0.703,1.104) | 0.881 (0.703,1.104) |
| Cardiac disorders | RCT | 1.954 (1.073,3.558) | 1.954 (1.073,3.558) |
| Mortality (Remdesivir with steroid) | Observational study | 0.777 (0.541,1.116) | 0.777 (0.541,1.116) |
| Duration of hospital stay  (Remdesivir with steroid) | Observational study | -1.774 (-5.283,1.736) | -1.774 (-5.283,1.736) |
| New admission to the ICU at baseline (Remdesivir with steroid) | Observational study | 0.921 (0.599,1.415) | 0.921 (0.599,1.415) |
| Liver injury (Remdesivir with steroid) (Remdesivir with steroid) | Observational study | 2.367 (0.222,25.194) | 1.000 (0.128,7.797) |
| Mortality (Remdesivir with tocilizumab) | Observational study | 2.034 (1.180,3.507) | 1.545 (0.947,2.521) |
| Mortality (Remdesivir with convalescent plasma) | Observational study | 1.424 (0.665,3.049) | 1.424 (0.665,3.049) |
| Mortality (Remdesivir with favipiravir) | Observational study | 0.984 (0.420,2.304) | 0.984 (0.420,2.304) |
