# Supplementary material for: The efficacy and safety of remdesivir alone and in combination with other drugs for the treatment of COVID-19: a systematic review and meta-analysis
Source: BMC Infect Dis. 2023 Oct 9;23:672. doi: 10.1186/s12879-023-08525-0 (PMC10563317; doi:10.1186/s12879-023-08525-0)
Supplement: Supplementary file 9 — Additional file 9. GRADE evidence Profiles. [file 12879_2023_8525_MOESM9_ESM.docx]

Additional file 9. GRADE evidence Profiles

| **Quality assessment** | | | | | | | **No of patients** | | **Effect** | | **Quality** | **Importance** |  |
| --- | --- | --- | --- | --- | --- | --- | --- | --- | --- | --- | --- | --- | --- |
|  |  |  |  |  |  |  |  |  |  |  |  |  |  |
| **No of studies** | **Design** | **Risk of bias** | **Inconsistency** | **Indirectness** | **Imprecision** | **Other considerations** | **Outcome** | **Control** | **Relative (95% CI)** | **Absolute** |  |  |  |
| **Mortality - Randomized controlled trials** | | | | | | | | | | | | |  |
| 7 | randomized trials | no serious risk of bias | no serious inconsistency | no serious indirectness | no serious imprecision | none | 433/4175  (10.4%) | 442/4060  (10.9%) | RR 0.94 (0.83 to 1.07) | 7 fewer per 1000 (from 19 fewer to 8 more) | ÅÅÅÅ HIGH | CRITICAL  (9) |  |
|  |  |  |  |  |  |  |  | 9.1% |  | 5 fewer per 1000 (from 15 fewer to 6 more) |  |  |  |
| **Mortality - Observational study** | | | | | | | | | | | | |  |
| 16 | observational studies | no serious risk of bias | no serious inconsistency^1^ | no serious indirectness | no serious imprecision | none | 475/4367  (10.9%) | 1203/9130  (13.2%) | RR 0.73 (0.59 to 0.9) | 36 fewer per 1000 (from 13 fewer to 54 fewer) | ÅÅOO LOW | CRITICAL  (9) |  |
|  |  |  |  |  |  |  |  | 13.8% |  | 37 fewer per 1000 (from 14 fewer to 57 fewer) |  |  |  |
| **Duration of hospital stay - Randomized controlled trials (Better indicated by lower values)** | | | | | | | | | | | | |  |
| 6 | randomized trials | serious^2^ | serious^3^ | no serious indirectness | no serious indirectness | none | 1881 | 1800 |  | MD 0.26 higher (2.45 lower to 2.97 higher) | ÅÅOO LOW | CRITICAL  (7) |  |
| **Duration of hospital stay - Observational study (Better indicated by lower values)** | | | | | | | | | | | | |  |
| 7 | observational studies | serious^2^ | serious^3^ | no serious indirectness | no serious indirectness | none | 1874 | 1997 |  | MD 1.23 lower (3.61 lower to 1.16 higher) | ÅOOO VERY LOW | CRITICAL  (7) |  |
| **Recovery - Randomized controlled trials** | | | | | | | | | | | | |  |
| 2 | randomized trials | no serious risk of bias | no serious inconsistency | no serious indirectness | no serious imprecision | none | 577/734  (78.6%) | 522/721  (72.4%) | RR 1.09 (1.03 to 1.15) | 65 more per 1000 (from 22 more to 109 more) | ÅÅÅÅ HIGH | CRITICAL  (7) |  |
|  |  |  |  |  |  |  |  | 76.3% |  | 69 more per 1000 (from 23 more to 114 more) |  |  |  |
| **Recovery- Observational study** | | | | | | | | | | | | |  |
| 4 | observational studies | no serious risk of bias | no serious inconsistency^4^ | no serious indirectness | no serious imprecision | none | 372/523  (71.1%) | 1017/1731  (58.8%) | RR 1.18 (1.05 to 1.32) | 106 more per 1000 (from 29 more to 188 more) | ÅÅOO LOW | CRITICAL  (7) |  |
|  |  |  |  |  |  |  |  | 69.3% |  | 125 more per 1000 (from 35 more to 222 more) |  |  |  |
| **Any adverse events - Randomized controlled trials** | | | | | | | | | | | | |  |
| 6 | randomized trials | no serious risk of bias | serious^3^ | no serious indirectness | no serious imprecision | none | 899/1607  (55.9%) | 855/1582  (54%) | RR 1.06 (0.93 to 1.2) | 32 more per 1000 (from 38 fewer to 108 more) | ÅÅÅO MODERATE | CRITICAL  (8) |  |
|  |  |  |  |  |  |  |  | 51.5% |  | 31 more per 1000 (from 36 fewer to 103 more) |  |  |  |
| **Serious adverse events - Randomized controlled trails** | | | | | | | | | | | | |  |
| 6 | randomized trials | no serious risk of bias | serious^3^ | no serious indirectness | no serious imprecision | none | 317/1607  (19.7%) | 363/1582  (22.9%) | RR 0.79 (0.60 to 1.0) | 49 fewer per 1000 (from 95 fewer to 9 more) | ÅÅÅO MODERATE | CRITICAL  (8) |  |
|  |  |  |  |  |  |  |  | 20.3% |  | 44 fewer per 1000 (from 86 fewer to 8 more) |  |  |  |
| **New use of mechanical ventilation or ECMO at baseline - Randomized controlled trails** | | | | | | | | | | | | |  |
| 6 | randomized trials | no serious risk of bias | serious^3^ | no serious indirectness | no serious imprecision | none | 389/3557  (10.9%) | 430/3519  (12.2%) | RR 0.78 (0.52 to 1.18) | 27 fewer per 1000 (from 59 fewer to 22 more) | ÅÅÅO MODERATE | IMPORTANT  (6) |  |
|  |  |  |  |  |  |  |  | 9.7% |  | 21 fewer per 1000 (from 47 fewer to 17 more) |  |  |  |
| **New use of mechanical ventilation or ECMO at baseline - Observational study** | | | | | | | | | | | | |  |
| 4 | observational studies | no serious risk of bias | serious^3^ | no serious indirectness | no serious imprecision | none | 77/818  (9.4%) | 264/2447  (10.8%) | RR 0.9 (0.56 to 1.47) | 11 fewer per 1000 (from 47 fewer to 51 more) | ÅOOO VERY LOW | IMPORTANT  (6) |  |
|  |  |  |  |  |  |  |  | 16.8% |  | 17 fewer per 1000 (from 74 fewer to 79 more) |  |  |  |
| **Days of mechanical ventilation or ECMO during study - Observational study (Better indicated by lower values)** | | | | | | | | | | | | |  |
| 2 | randomized trials | serious^2^ | serious^3^ | no serious indirectness | no serious imprecision | none | 699 | 599 | - | MD 0.36 higher (2.38 lower to 3.1 higher) | ÅÅOO LOW | IMPORTANT  (6) |  |
| **New use of noninvasive ventilation or high-flow oxygen at baseline - Randomized controlled trials** | | | | | | | | | | | | |  |
| 3 | randomized trials | no serious risk of bias | no serious inconsistency^4^ | no serious indirectness | no serious imprecision | none | 71/534  (13.3%) | 93/502  (18.5%) | RR 0.75 (0.48 to 1.18) | 46 fewer per 1000 (from 96 fewer to 33 more) | ÅÅÅÅ HIGH | IMPORTANT  (6) |  |
|  |  |  |  |  |  |  |  | 24.1% |  | 38 fewer per 1000 (from 86 fewer to 42 more) |  |  |  |
| **Days to negative PCR - Observational study (Better indicated by lower values)** | | | | | | | | | | | | |  |
| 2 | observational studies | no serious risk of bias | serious^3^ | no serious indirectness | no serious imprecision | none | 339 | 144 | - | MD 0.8 lower (5.3 lower to 3.7 higher) | ÅOOO VERY LOW | IMPORTANT  (4) |  |
| **New use of oxygen or low-flow oxygen at baseline - Randomized controlled trials** | | | | | | | | | | | | |  |
| 4 | randomized trials | no serious risk of bias | no serious inconsistency | no serious indirectness | no serious imprecision | none | 59/373  (15.8%) | 61/353  (17.3%) | RR 0.83 (0.61 to 1.12) | 29 fewer per 1000 (from 67 fewer to 21 more) | ÅÅÅÅ HIGH | IMPORTANT  (5) |  |
|  |  |  |  |  |  |  |  | 23.2% |  | 39 fewer per 1000 (from 90 fewer to 28 more) |  |  |  |
| **New use of oxygen or low-flow oxygen at baseline - Observational study** | | | | | | | | | | | | |  |
| 2 | observational studies | no serious risk of bias | no serious inconsistency | no serious indirectness | no serious imprecision | none | 307/332  (92.5%) | 121/240  (50.4%) | RR 1.72 (1.48 to 2) | 363 more per 1000 (from 242 more to 504 more) | ÅÅOO LOW | IMPORTANT  (5) |  |
|  |  |  |  |  |  |  |  | 50.8% |  | 366 more per 1000 (from 244 more to 508 more) |  |  |  |
| **Days of receiving oxygen or low-flow oxygen during study - Randomzied controlled trails (Better indicated by lower values)** | | | | | | | | | | | | |  |
| 2 | randomized trials | serious^2^ | no serious inconsistency | no serious indirectness | no serious imprecision | none | 699 | 599 | - | MD 0.24 higher (2.57 lower to 3.05 higher) | ÅÅÅO MODERATE | IMPORTANT  (5) |  |
| **New admission to the ICU at baseline - Observational study** | | | | | | | | | | | | |  |
| 2 | observational studies | no serious risk of bias | no serious inconsistency | no serious indirectness | no serious imprecision | none | 124/476  (26.1%) | 405/2296  (17.6%) | RR 1.46 (1.23 to 1.74) | 81 more per 1000 (from 41 more to 131 more) | ÅÅOO LOW | IMPORTANT  (5) |  |
|  |  |  |  |  |  |  |  | 22.3% |  | 103 more per 1000 (from 51 more to 165 more) |  |  |  |
| **Clinical improvement - Randomized controlled trails** | | | | | | | | | | | | |  |
| 2 | randomized trials | no serious risk of bias | no serious inconsistency | no serious indirectness | no serious imprecision | none | 277/351  (78.9%) | 211/278  (75.9%) | RR 1.09 (1.01 to 1.17) | 68 more per 1000 (from 8 more to 129 more) | ÅÅÅÅ HIGH | IMPORTANT  (5) |  |
|  |  |  |  |  |  |  |  | 70.4% |  | 63 more per 1000 (from 7 more to 120 more) |  |  |  |
| **Clinical improvement - Observational study** | | | | | | | | | | | | |  |
| 3 | observational studies | no serious risk of bias | no serious inconsistency^4^ | no serious indirectness | no serious imprecision | none | 665/737  (90.2%) | 1434/1732  (82.8%) | RR 1.09 (1.01 to 1.18) | 75 more per 1000 (from 8 more to 149 more) | ÅÅOO LOW | IMPORTANT  (5) |  |
|  |  |  |  |  |  |  |  | 84% |  | 76 more per 1000 (from 8 more to 151 more) |  |  |  |
| **Time to clinical improvement - Randomized controlled trials (Better indicated by lower values)** | | | | | | | | | | | | |  |
| 3 | randomized trials | serious^2^ | no serious inconsistency | no serious indirectness | no serious imprecision | none | 1113 | 1017 | - | MD 2.51 lower (2.75 to 2.28 lower) | ÅÅÅO MODERATE | IMPORTANT  (5) |  |
| **Time to recovery - Observational study (Better indicated by lower values)** | | | | | | | | | | | | |  |
| 2 | observational studies | serious^2^ | no serious inconsistency | no serious indirectness | serious^3^ | none | 147 | 163 | - | MD 0.92 lower (1.84 to 0.01 lower) | ÅOOO VERY LOW | IMPORTANT  (5) |  |
| **Discharge - Randomized controlled trails** | | | | | | | | | | | | |  |
| 2 | randomized trials | no serious risk of bias | no serious inconsistency | no serious indirectness | no serious imprecision | none | 266/343  (77.6%) | 211/277  (76.2%) | RR 1.08 (1.01 to 1.17) | 61 more per 1000 (from 8 more to 129 more) | ÅÅÅÅ HIGH | IMPORTANT  (5) |  |
|  |  |  |  |  |  |  |  | 70.7% |  | 57 more per 1000 (from 7 more to 120 more) |  |  |  |
| **Discharge - Observational study** | | | | | | | | | | | | |  |
| 4 | observational studies | no serious risk of bias | serious^3^ | no serious indirectness | no serious imprecision | none | 487/533  (91.4%) | 1257/1565  (80.3%) | RR 1.14 (1.02 to 1.28) | 112 more per 1000 (from 16 more to 225 more) | ÅOOO VERY LOW | IMPORTANT  (5) |  |
|  |  |  |  |  |  |  |  | 74.8% |  | 105 more per 1000 (from 15 more to 209 more) |  |  |  |
| **Kidney injury - Randomized controlled trails** | | | | | | | | | | | | |  |
| 6 | randomized trials | no serious risk of bias | no serious inconsistency | no serious indirectness | no serious imprecision | none | 102/1945  (5.2%) | 119/1929  (6.2%) | RR 0.87 (0.68 to 1.11) | 8 fewer per 1000 (from 20 fewer to 7 more) | ÅÅÅÅ HIGH | CRITICAL  (8) |  |
|  |  |  |  |  |  |  |  | 3% |  |  |  |  |  |
| **Kidney injury - Observational study** | | | | | | | | | | | | |  |
| 3 | observational studies | no serious risk of bias | serious^3^ | no serious indirectness | no serious imprecision | none | 55/231  (23.8%) | 135/408  (33.1%) | RR 0.77 (0.41 to 1.45) | 76 fewer per 1000 (from 195 fewer to 149 more) | ÅOOO VERY LOW | CRITICAL  (8) |  |
|  |  |  |  |  |  |  |  | 29.5% |  | 68 fewer per 1000 (from 174 fewer to 133 more) |  |  |  |
| **Liver injury - Randomized controlled trials** | | | | | | | | | | | | |  |
| 6 | randomized trials | no serious risk of bias | no serious inconsistency | no serious indirectness | no serious imprecision | none | 160/1946  (8.2%) | 178/1928  (9.2%) | RR 0.88 (0.7 to 1.11) | 11 fewer per 1000 (from 28 fewer to 10 more) | ÅÅÅÅ HIGH | CRITICAL  (8) |  |
|  |  |  |  |  |  |  |  | 6.5% |  | 8 fewer per 1000 (from 20 fewer to 7 more) |  |  |  |
|  |  |  |  |  |  |  |  | 27.2% |  | 82 more per 1000 (from 33 fewer to 250 more) |  |  |  |
| **Cardiac disorders - Randomized controlled trials** | | | | | | | | | | | | |  |
| 4 | randomized trials | no serious risk of bias | no serious inconsistency | no serious indirectness | no serious imprecision | none | 31/1135  (2.7%) | 16/1099  (1.5%) | RR 1.95 (1.07 to 3.56) | 14 more per 1000 (from 1 more to 37 more) | ÅÅÅÅ HIGH | CRITICAL  (8) |  |
|  |  |  |  |  |  |  |  | 1.4% |  | 13 more per 1000 (from 1 more to 36 more) |  |  |  |
|  |  |  |  |  |  |  |  | 17.3% |  | 61 more per 1000 (from 31 fewer to 213 more) |  |  |  |
| **Mortality - Observational study (Remdesivir with steroid)** | | | | | | | | | | | | |  |
| 6 | observational studies | no serious risk of bias | serious^3^ | no serious indirectness | no serious imprecision | none | 466/2975  (15.7%) | 472/2305  (20.5%) | RR 0.78 (0.54 to 1.12) | 45 fewer per 1000 (from 94 fewer to 25 more) | ÅOOO VERY LOW | CRITICAL  (9) |  |
|  |  |  |  |  |  |  |  | 18.7% |  | 41 fewer per 1000 (from 86 fewer to 22 more) |  |  |  |
| **Duration of hospital stay- Observational study (Remdesivir with steroid) (Better indicated by lower values)** | | | | | | | | | | | | |  |
| 4 | observational studies | serious^2^ | serious^3^ | no serious indirectness | no serious imprecision | none | 1902 | 1232 | - | MD 1.77 lower (5.28 lower to 1.74 higher) | ÅOOO VERY LOW | CRITICAL  (7) |  |
| **New admission to the ICU at baseline - Observational study (Remdesivir with steroid)** | | | | | | | | | | | | |  |
| 3 | observational studies | no serious risk of bias | serious^3^ | no serious indirectness | no serious imprecision | none | 283/1131  (25%) | 244/1103  (22.1%) | RR 0.92 (0.6 to 1.41) | 18 fewer per 1000 (from 88 fewer to 91 more) | ÅOOO VERY LOW | IMPORTANT  (5) |  |
|  |  |  |  |  |  |  |  | 22.2% |  | 18 fewer per 1000 (from 89 fewer to 91 more) |  |  |  |
| **Liver injury - Observational study (Remdesivir with steroid)** | | | | | | | | | | | | |  |
| 2 | observational studies | no serious risk of bias | serious^3^ | no serious indirectness | no serious imprecision | none | 146/1075  (13.6%) | 140/1074  (13%) | RR 2.39 (0.22 to 26.12) | 181 more per 1000 (from 102 fewer to 1000 more) | ÅOOO VERY LOW | CRITICAL  (8) |  |
|  |  |  |  |  |  |  |  | 7% |  | 97 more per 1000 (from 55 fewer to 1000 more) |  |  |  |
|  |  |  |  |  |  |  |  | 19.5% |  | 14 fewer per 1000 (from 66 fewer to 60 more) |  |  |  |
| **Mortality - Observational study (Remdesivir with tocilizumab)** | | | | | | | | | | | | |  |
| 3 | observational studies | no serious risk of bias | no serious inconsistency ^4^ | no serious indirectness | no serious imprecision | strong association^6^ | 60/134  (44.8%) | 153/528  (29%) | RR 2.05 (1.17 to 3.61) | 304 more per 1000 (from 49 more to 756 more) | ÅÅÅO MODERATE | CRITICAL  (9) |  |
|  |  |  |  |  |  |  |  | 14.7% |  | 154 more per 1000 (from 25 more to 384 more) |  |  |  |
| **Mortality - Observational study (Remdesivir with convalescent plasma)** | | | | | | | | | | | | |  |
| 4 | observational studies | no serious risk of bias | serious^3^ | no serious indirectness | no serious imprecision | none | 71/202  (35.1%) | 163/996  (16.4%) | RR 1.42 (0.66 to 3.07) | 69 more per 1000 (from 56 fewer to 339 more) | ÅOOO VERY LOW | CRITICAL  (9) |  |
|  |  |  |  |  |  |  |  | 21.7% |  | 91 more per 1000 (from 74 fewer to 449 more) |  |  |  |
| **Mortality - Observational study (Remdesivir with favipiravir)** | | | | | | | | | | | | |  |
| 2 | observational studies | no serious risk of bias | no serious inconsistency | no serious indirectness | serious^5^ | none | 11/31  (35.5%) | 21/78  (26.9%) | RR 0.98 (0.42 to 2.32) | 5 fewer per 1000 (from 156 fewer to 355 more) | ÅOOO VERY LOW | CRITICAL  (9) |  |
|  |  |  |  |  |  |  |  | 42.3% |  | 8 fewer per 1000 (from 245 fewer to 558 more) |  |  |  |
| *The basis for the **assumed risk** (e.g. the median control group risk across studies) is provided in footnotes. The **corresponding risk** (and its 95% confidence interval) is based on the assumed risk in the comparison group and the **relative effect** of the intervention (and its 95% CI). **CI:** Confidence interval; **RR:** Risk ratio; | | | | | | | | | | | | |  |
| GRADE Working Group grades of evidence **High quality:** Further research is very unlikely to change our confidence in the estimate of effect.  **Moderate quality:** Further research is likely to have an important impact on our confidence in the estimate of effect and may change the estimate. **Low quality:** Further research is very likely to have an important impact on our confidence in the estimate of effect and is likely to change the estimate. **Very low quality:** We are very uncertain about the estimate. | | | | | | | | | | | | |  |
| ^1^ There is heterogeneity, but meta-regression analysis and subgroup analysis have found the source of heterogeneity.  ^2^ Data is transformed by formula  ^3^ Outcome showed heterogeneity and unexplained  ^4^ There was heterogeneity, but sensitivity analysis found the source of heterogeneity and heterogeneity is dismissed.  ^5^ The sample size of the included studies is too small  ^6^ There were two studies with RR > 2 | | | | | | | | | | | | |  |
